# ALIX and TSG101 are essential for cellular entry and replication of two porcine alphacoronaviruses

**DOI:** 10.1371/journal.ppat.1012103

**Published:** 2024-03-15

**Authors:** Xiongnan Chen, Yifan Liang, Zhijun Weng, Chen Hu, Yunzhao Peng, Yingshuo Sun, Qi Gao, Zhao Huang, Shengqiu Tang, Lang Gong, Guihong Zhang

**Affiliations:** 1 Guangdong Provincial Key Laboratory of Zoonosis Prevention and Control, College of Veterinary Medicine, South China Agricultural University, Guangzhou, China; 2 Guangdong Provincial Key Laboratory of Utilization and Conservation of Food and Medicinal Resources in Northern Region, Shaoguan University, Shaoguan, China; 3 Maoming Branch, Guangdong Laboratory for Lingnan Modern Agriculture, Guangdong, China; 4 Key Laboratory of Animal Vaccine Development, Ministry of Agriculture and Rural Affairs, China; University of Maryland at College Park: University of Maryland, UNITED STATES

## Abstract

Alphacoronaviruses are the primary coronaviruses responsible for causing severe economic losses in the pig industry with the potential to cause human outbreaks. Currently, extensive studies have reported the essential role of endosomal sorting and transport complexes (ESCRT) in the life cycle of enveloped viruses. However, very little information is available about which ESCRT components are crucial for alphacoronaviruses infection. By using RNA interference in combination with Co-immunoprecipitation, as well as fluorescence and electron microscopy approaches, we have dissected the role of ALIX and TSG101 for two porcine alphacoronavirus cellular entry and replication. Results show that infection by two porcine alphacoronaviruses, including porcine epidemic diarrhea virus (PEDV) and porcine enteric alphacoronavirus (PEAV), is dramatically decreased in ALIX- or TSG101-depleted cells. Furthermore, PEDV entry significantly increases the interaction of ALIX with caveolin-1 (CAV1) and RAB7, which are crucial for viral endocytosis and lysosomal transport, however, does not require TSG101. Interestingly, PEAV not only relies on ALIX to regulate viral endocytosis and lysosomal transport, but also requires TSG101 to regulate macropinocytosis. Besides, ALIX and TSG101 are recruited to the replication sites of PEDV and PEAV where they become localized within the endoplasmic reticulum and virus-induced double-membrane vesicles. PEDV and PEAV replication were significantly inhibited by depletion of ALIX and TSG101 in Vero cells or primary jejunal epithelial cells, indicating that ALIX and TSG101 are crucial for PEDV and PEAV replication. Collectively, these data highlight the dual role of ALIX and TSG101 in the entry and replication of two porcine alphacoronaviruses. Thus, ESCRT proteins could serve as therapeutic targets against two porcine alphacoronaviruses infection.

## Author summary

Alphacoronaviruses are one of the main coronaviruses that cause infections in humans and animals. However, effective antivirals to protect against alphacoronavirus infections are lacking. It is, therefore, necessary to better understand the interactions between these viruses and host cells to inform the development of improved preventatives and therapeutics. Here we assessed the role of the ESCRT system—responsible for viral lifecycle—in the common porcine epidemic diarrhea virus (PEDV) and the emerging highly pathogenic porcine enteric alphacoronavirus (PEAV). In particular, the roles of ALIX, and TSG101 were evaluated in the entry and replication of PEDV and PEAV. The results indicate that ALIX and TSG101 regulate viral infection by inducing host cell-mediated endocytosis and altering viral transport dynamics in the endosome/lysosomal system. In addition, both ESCRT proteins have important roles in mediating the formation of viral replication organelles. Hence, ALIX and TSG101 may represent host proteins that can act as targets of antiviral therapies to protect animals and humans against alphacoronavirus infection.

## Introduction

The alphacoronaviruses includes porcine epidemic diarrhea virus (PEDV), transmissible gastroenteritis virus (TGEV), swine diarrhea syndrome virus (SDS-CoV/PEAV), human coronaviruses (HCoV-NL63 and HCoV-229E), and cat coronavirus (FCoV). Among the porcine diarrheal viruses, PEDV is the most harmful, causing huge economic losses to the global pig industry [1]. Meanwhile, PEAV is a newly discovered porcine diarrheal virus, identified as a new bat-derived coronavirus related to HKU2 [2], and is the first coronavirus to spread from bats to pigs [3]. It has a wide range of cellular preferences and may have the ability to be transmitted across species [4,5]. However, currently, the receptors are still unknown, and no vaccines or specific antiviral drugs are available for these two types of alphacoronaviruses.

Alphacoronaviruses are positive-stranded RNA viruses. After their internalization by host cells, they are transported to the replication site to release genomic RNA. The viral genome encodes two polyproteins (ORF1a and ORF1ab) that become proteolytically cleaved via virus-encoded proteases into 16 nonstructural proteins (NSPs), 4 structural proteins: [spike (S), envelope (E), membrane (M), and nucleocapsid (N)] and 1 accessory proteins, open reading frame (ORF)3a. Unlike PEDV, PEAV additionally contains NSP7A and NSP7B. Although the virulence and immune escape mechanisms of various coronaviruses become altered via amino acid mutation, addition, or deletion, certain processes are highly conserved, including virus endocytosis, replication of organelle formation, virus assembly, and budding. Accordingly, these process represent promising targets for broad-spectrum antiviral therapies.

Many enveloped viruses typically require the recruitment of conserved host endocytosis transport or membrane rearrangement machinery to complete their lifecycle. In particular, the ESCRT system is conserved in eukaryotes and comprises several heteropolymer complexes. ESCRT-0 primarily recognizes ubiquitinated proteins, ESCRT-I (TSG101), ESCRT-II, and ALIX are responsible for recruiting and inducing membrane deformation, and ESCRT-III contributes to membrane fission. TSG101 and ALIX also initiate two parallel ESCRT-III nucleation and assembly pathways during the assembly of ECSRT complexes [6–8]. Considering that ESCRT has many functions, including endosomal maturation, autophagy, and membrane protein sorting, its topological structure pattern is similar to that of replication organelle formation, virus assembly, and budding processes [9]. Coronavirus infection is closely related to endosomal maturation and autophagy [10,11]. In addition, its replication requires the formation of replication organelle, double membrane vesicles (DMVs) [12], therefore, we speculated that alphacoronaviruses require ESCRT proteins for their infection cycle.

In this study, we investigated the roles of ALIX and TSG101 in two porcine alphacoronaviruses infection in primary and immortalized susceptible cell lines. We discovered that PEDV or PEAV infection was greatly suppressed in ALIX- or TSG101-depleted cells. ALIX was recruited to caveolin-1 (CAV1) or Rab7-positiveendosome for viral endocytosis and lysosomal transport. TSG101 was recruited to promote viral macropinocytosis and subsequent lysosomal transport. We also demonstrated that although TSG101 is not necessary for PEDV entry, both ALIX and TSG101 are crucial for viral replication. ALIX and TSG101 are simultaneously located in DMVs, indicating that ALIX and TSG101 play dual roles in the entry and replication of two porcine alphacoronaviruses. Our findings provide insights into the novel role of ESCRT for alphacoronavirus entry and replication.

## Results

### ALIX and TSG101 are critical for PEDV/PEAV infection

**Fig 1:**
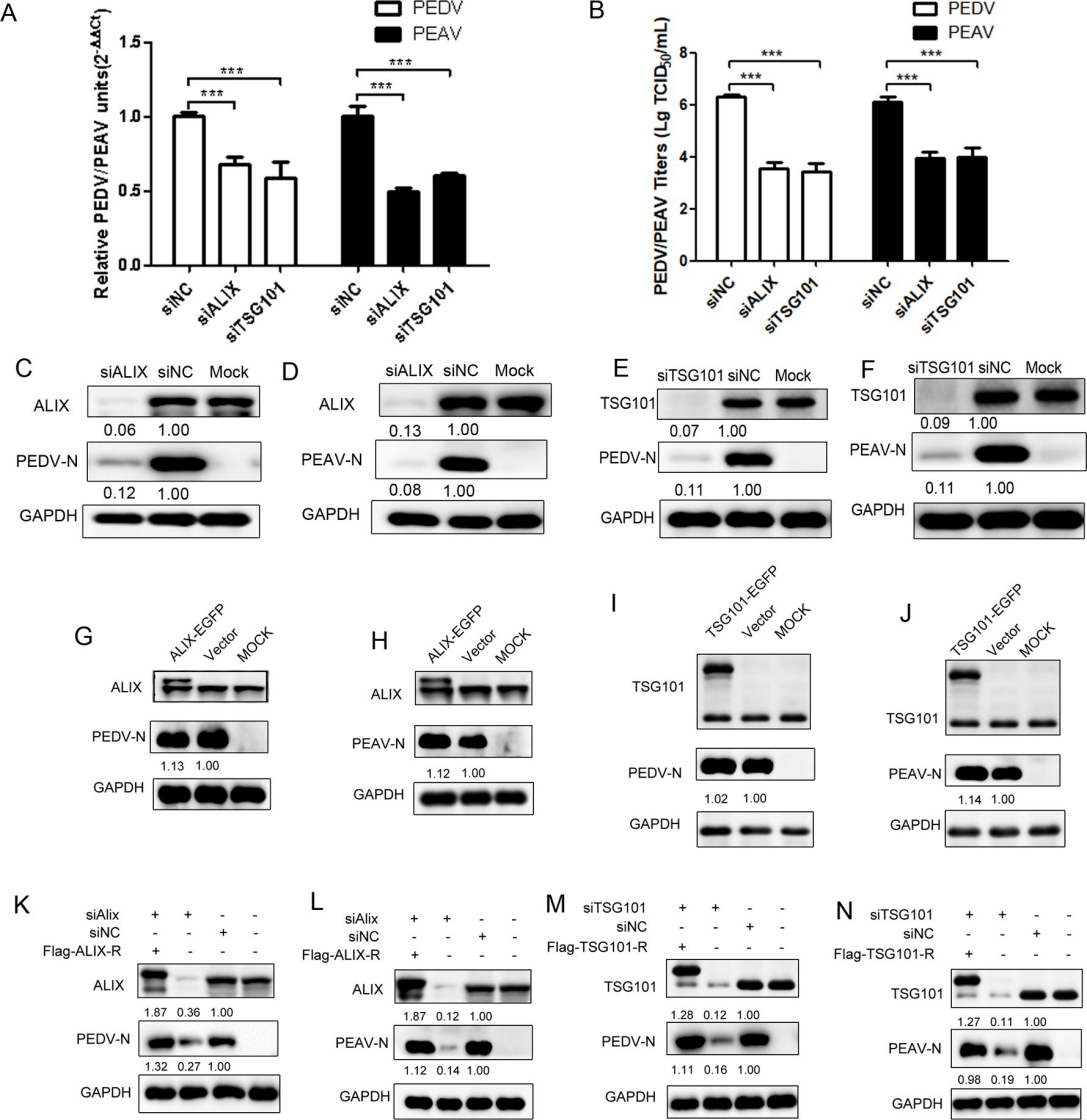
ALIX and TSG101 is required for PEDV/PEAV infection. (A) PEDV or PEAV RNA levels in Vero cells transfected with siALIX, siTSG101, or siCtrl for 48 h and infected with PEDV or PEAV (MOI = 0.1, 24 h). (B) PEDV or PEAV titers in Vero cells transfected with siALIX, siTSG101, or siCtrl. (C and D) ALIX, (E and F) TSG101, and PEDV or PEAV N protein abundance in Vero cells transfected with siALIX, siTSG101, or siCtrl for 48 h and infected with PEDV or PEAV (MOI = 0.1, 24 h). GAPDH is an internal control. (G-J) Vero cells transfected with plasmids for (G and H) EGFP-ALIX or (I and J) EGFP-TSG101, or vector for 6 h, then infected with PEDV or PEAV (MOI = 0.1, 18 h). (K-N) Plasmids expressing (K and L) ALIX or (M and N) TSG101 proteins with siRNA-resistant silent mutations were co-transfected with siRNAs for 48 h and infected with PEDV or PEAV (MOI = 0.1, 24 h), and monitored for phenotype rescue by the measurement of viral N protein abundance. All results are presented as the mean ± SD from three independent experiments (*** *P* < 0.001).

An increasing number of studies have shown that ESCRT is involved in the life cycle of retroviruses [13], flaviviruses [14], and herpes viruses [15]. These viruses recruit ESCRT proteins by encoding conserved amino acid motifs, such as (YPX (n)L) [16] and PT/SAP [17]. To evaluate the roles of ALIX and TSG101 in PEDV or PEAV infection, we depleted ALIX and TSG101 in Vero cells and monitored their propagation. Compared with the control siRNA treatment, ALIX or TSG101 knockdown significantly decreased viral RNA levels in cells (Fig 1A). After infection, the cells were collected for viral titer testing. The results showed that lower levels of infectious particles were detected in cells depleted of ALIX or TSG101 compared to the control siRNA treatment (Fig 1B). Similarly, following ALIX (Fig 1C and 1D) or TSG101 (Fig 1E and 1F) depletion, PEDV and PEAV N protein expression was significantly reduced (Fig 1C–1F). Western blot assay showed that Vero cells overexpressing ALIX or TSG101 had an equivalent PEDV or PEAV N expression levels compared to control cells (Fig 1G–1J). However, the reduction of PEDV or PEAV N expression levels in ALIX or TSG101-depleted cells was rescued by exogenous expression of ALIX or TSG101 with silent mutations in the siRNA target sequence (Fig 1K–1N). Hence, ALIX and TSG101 might have important roles in PEDV and PEAV infection.

### The roles of ALIX and TSG101 in the entry of two porcine alphacoronaviruses

**Fig 2:**
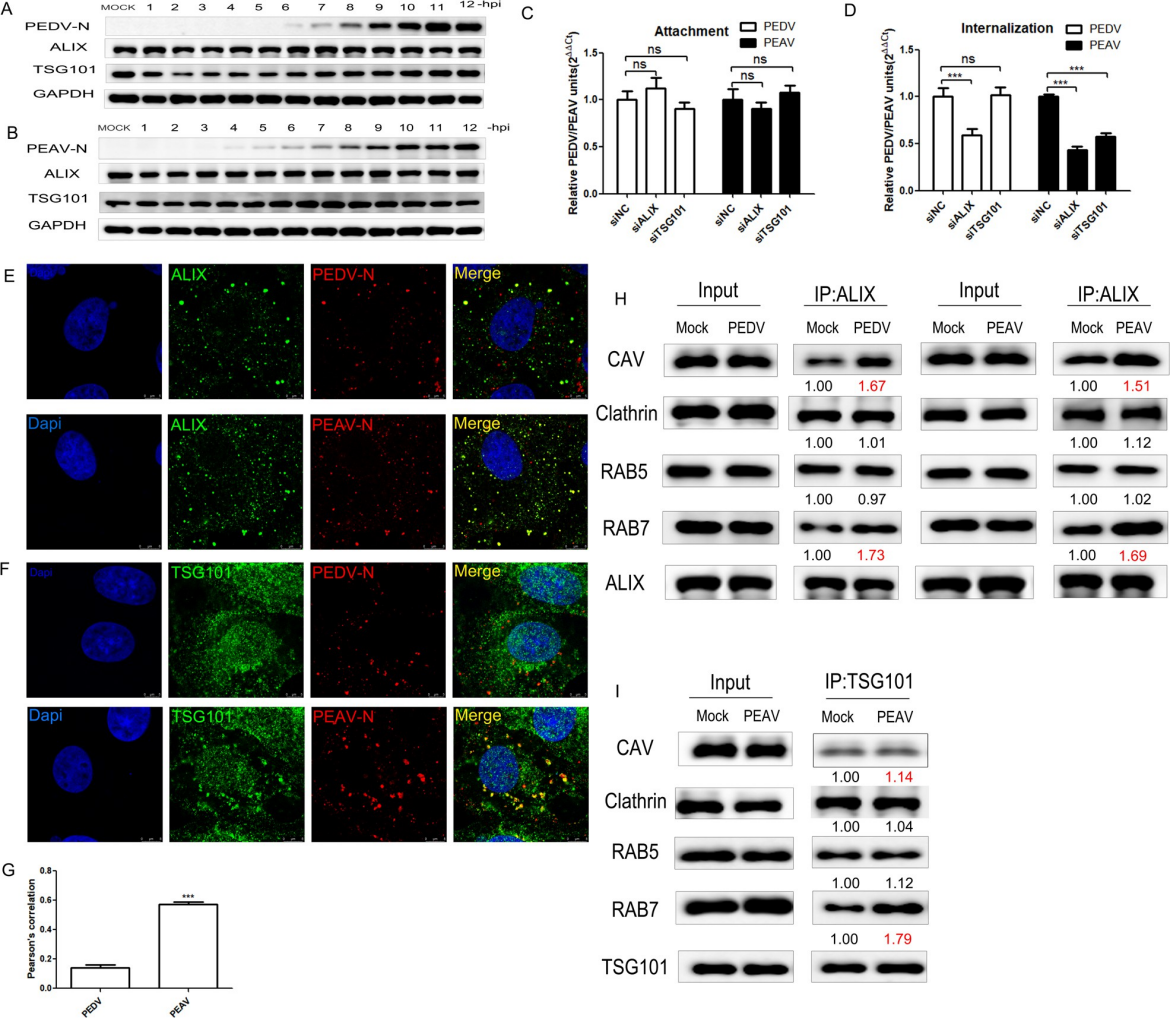
PEDV entry dependent on ALIX, while PEAV entry requires ALIX and TSG101. (A and B) (A) PEDV or (B) PEAV N protein, ALIX, and TSG101 abundance in Vero cells infected with PEDV or PEAV (MOI = 0.5) and harvested at 1 h intervals over 12 h (one replication cycle). GAPDH is the internal control. (C and D) (C) Attachment (1 h, 4°C) (D) internalization (1 h,37°C) of PEDV or PEAV by Vero cells transfected with siALIX, siTSG101 or siCtrl for 48 h before PEDV infection (Cells were washed with PBS and treated with proteinase K (0.5 mg/ml) for 5 min at 4°C to remove adsorbed but not internalized virus). (E and F) Vero cells infected with PEDV or PEAV (MOI = 20, 30 min); red: anti-PEDV or PEAV N, green: (E) anti-ALIX or (F) anti-TSG101; Scale bar = 8 μm. (G) Co-localization of TSG101 with PEDV or PEAV N expressed as Pearson’s correlation coefficient, measured for individual cells. (H and I) Vero cells were infected with PEDV or PEAV (MOI = 20) or not for 30 min, then harvested for immunoprecipitation by using rabbit anti-ALIX antibody (H) or rabbit anti-TSG101 (I), and the whole-cell lysates were subjected to Western blotting by using rabbit anti-CAV1, RAB5, RAB7 (Proteintech, USA), mouse anti-ALIX, and TSG101. These data are representative of three independent experiments. All results are presented as the mean ± SD from three independent experiments (*** *P* < 0.001; ns, P > 0.05).

To determine the stages of PEDV infection that ALIX and TSG101 are involved in, Vero cells were collected at different infection times, and endogenous ALIX and TSG101 expression levels were determined. PEDV and PEAV infection did not significantly upregulate or downregulate endogenous ALIX or TSG101 expression (Fig 2A and 2B). Hence, PEDV infection did not directly affect viral propagation by regulating ALIX and TSG101 expression.

Next, we assessed the effect of ALIX or TSG101 depletion on viral entry. Compared to the control siRNA treatment, the depletion of ALIX or TSG101 did not impact the adsorption of PEDV and PEAV (Fig 2C). However, the depletion of ALIX significantly diminished the internalization of PEDV and PEAV, while the depletion of TSG101 had no effect on PEDV internalization but notably reduced PEAV internalization (Fig 2D). To further verify the involvement of ALIX in PEDV and PEAV entry, confocal microscopy was used to observe the localization of the virus and endogenous ALIX in PEDV or PEAV-infected Vero cells. After 30 min of infection, PEDV and PEAV co-localized with endogenous ALIX (Fig 2E). Nevertheless, only PEAV and TSG101 displayed noteworthy co-localization, whereas PEDV and TSG101 did not demonstrate significant co-localization (Fig 2F). This is consistent with the experimental results of inoculating cells with VLPs (Virus-like particles) induced by viral structural proteins (S1A–S1C Fig). Pearson’s correlation analysis revealed that the co-localization coefficient between TSG101 and PEAV or PEAV VLPs was markedly higher than that of PEDV or PEDV VLPs (Figs 2G and S1D). Hence, ALIX is indispensable for the entry of both PEDV and PEAV, whereas TSG101 is specifically essential for the entry of PEAV.

Next, considering that PEDV has been shown to mediate viral endocytosis through CAV1 and Clathrin, with RAB5 and RAB7 regulating endosome transport after viral internalization [18,19], we employed immunoprecipitation experiments to explore their interactions with CAV1, Clathrin, RAB5, and RAB7. Our results revealed ALIX’s interactions with CAV1, RAB7 were increased after PEDV or PEAV infection (Fig 2H). Additionally, TSG101’s interactions with RAB7 were increased after PEAV infection (Fig 2I). This suggests that PEDV and PEAV might mediate virus internalization by recruiting ALIX to CAV1, while requiring ALIX to complete the late endosome transport step. Nevertheless, the internalization of PEAV also necessitates the involvement of TSG101, which is essential for the completion of the endosome transport step.

### The roles of ALIX and TSG101 in the transport from late endosomes to lysosomes of two porcine alphacoronaviruses

**Fig 3:**
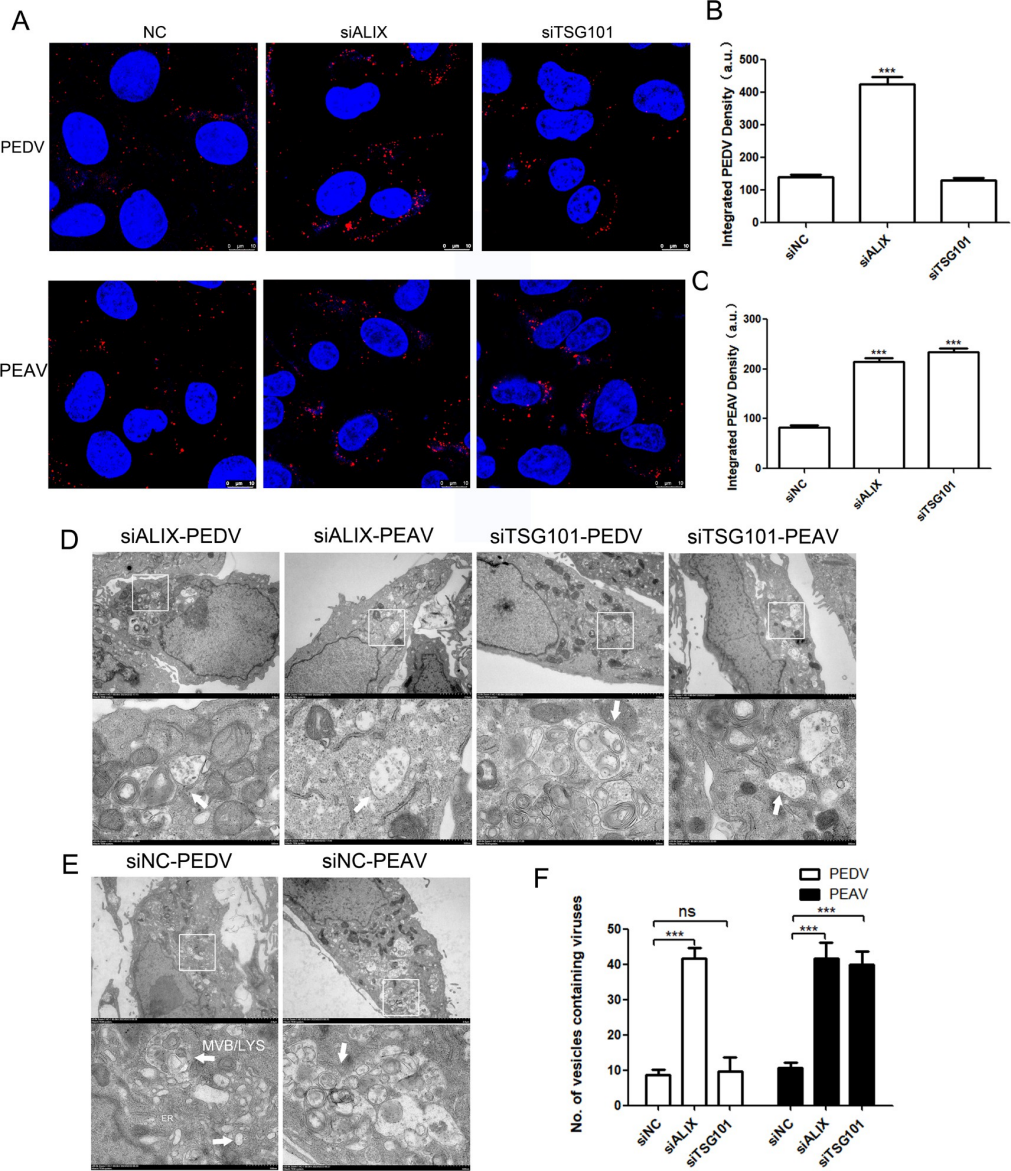
Different requirements for ALIX and TSG101 in the lysosomal transport processes of PEDV and PEAV. Vero cells transfected with (A-C) (A) siCTrl, siALIX, or siTSG101 for 48 h, then inoculate with Alexa Fluor 647 NHS ester-labeled PEDV or PEAV for 2 h (MOI = 20); scale bar = 10 μm. (B and C) More than 300 cells were included in the quantitative fluorescence density through image J 7.0 software. (D and E) Transmission electron microscopy analysis of the intracellular transport characteristics of PEDV or PEAV in Vero cells transfected with siALIX, siTSG101, or siCtrl for 48 h and infected with PEDV or PEAV (MOI = 50, 2 h). (F) Quantification of vesicles containing viruses numbers per 60 cells is shown as mean ± SD. All results are presented as the mean ± SD from three independent experiments (***, *P* < 0.001; ns, P > 0.05).

To determine whether ALIX or TSG101 is involved in the late endosome transport step of PEDV and PEAV, we labeled PEDV particles with fluorescent dyes. During the late endosome transport step, the virus undergoes membrane fusion and completes cytoplasmic release of the viral genome. This process leads to fluorescence labeling on the surface of the viral membrane and late endosome/lysosome membrane fusion, and the fluorescence is quenched or lower than the detection threshold of confocal microscopy [19]. Therefore, by observing the degree of fluorescence quenching, we determined whether PEDV or PEAV completed the endosome transport step. The results showed that, compared to the control siRNA treatment, a large number of fluorescently-labeled PEDVs or PEAVs were observed after ALIX depletion. Conversely, after TSG101 depletion, the quantity of fluorescence-labeled viruses in the PEAV group was markedly higher than that in the PEDV group, with the fluorescence density in the PEDV group being comparable to that of the control group (Fig 3A–3C).

To illustrate the role of ALIX and TSG101 in transporting PEDV or PEAV, we depleted ALIX or TSG101 in Vero cells and visualized cells using transmission electron microscopy 2 h after infection. In the control siRNA treatment group, many membrane structures appeared in the cells, indicating that the virus completed the cytoplasmic release process of the viral genome, entered the early replication stage, and induced membrane rearrangement to form organelles for viral replication. However, large amounts of PEDV accumulated in the endosomes of ALIX-depleted cells. Similar to the control group, fusion occurred between late endosomes and lysosomes after depletion of TSG101, resulting in degraded viral particle fragments (Fig 3D–3E). The failure of membrane fusion implies an increased presence of virus-containing endosomes within the cells. Employing a method of quantification outlined in the supplementary information (S2A–S2D Fig), we tallied the number of endosomes containing PEDV or PEAV in infected cells. Our findings revealed that following ALIX depletion, the abundance of endosomes containing PEDV or PEAV was significantly higher than in the control group. Moreover, the depletion of TSG101 only resulted in a higher number of virus-containing endosomes in the PEAV-infected group than in the control group, while that in the PEDV group was similar to the control group (Fig 3F). Hence, the lysosomal trafficking of PEDV and PEAV requires ALIX, while TSG101 is essential for PEAV but not mandatory for PEDV.

### The role of TSG101 in PEAV macropinocytosis

**Fig 4:**
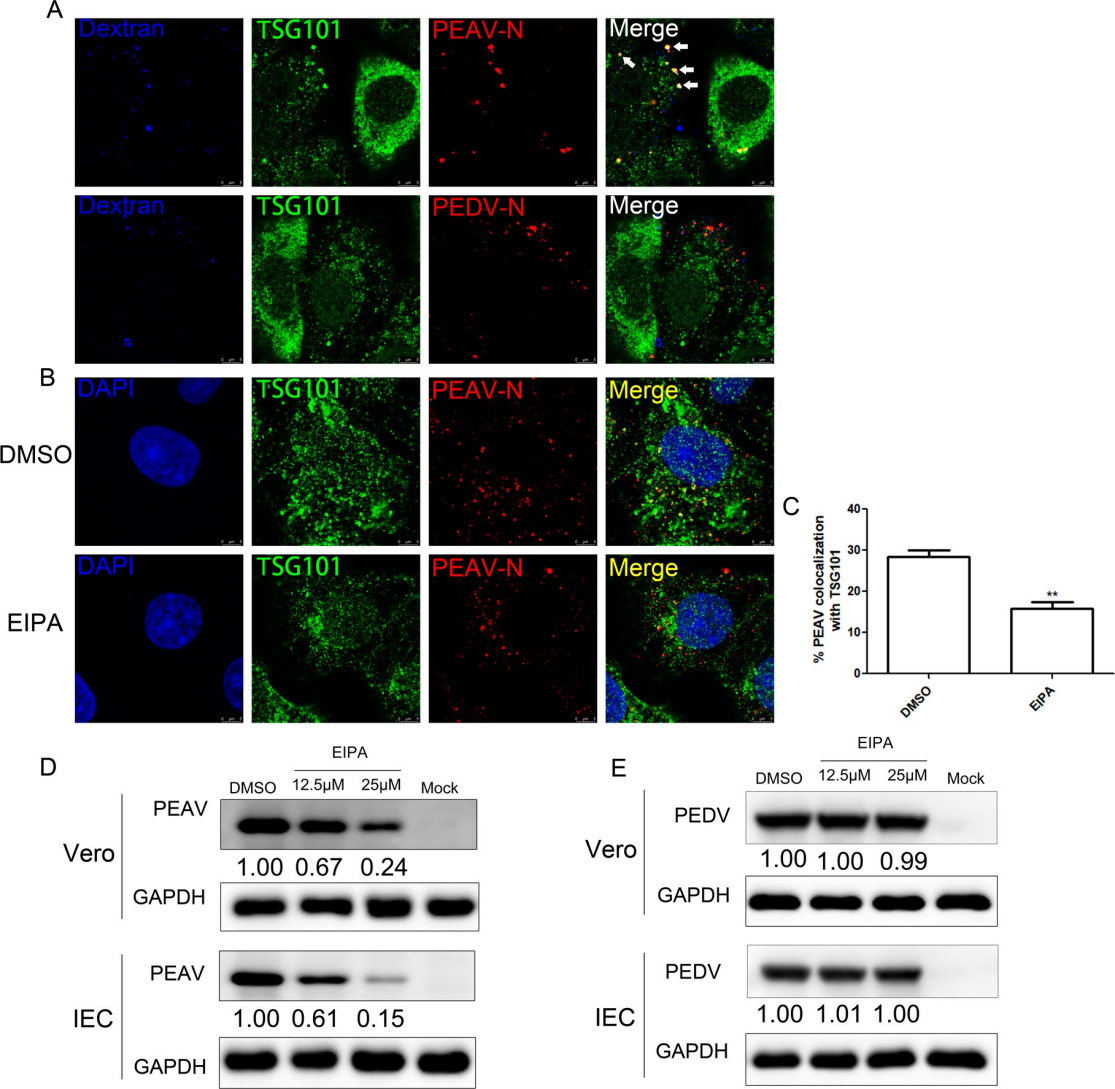
TSG101 is associated with PEAV macropinocytosis. (A) Vero cells inoculated with PEDV or PEAV (MOI = 20, 30 min); red: anti-PEAV N, green: anti-TSG101, blue: dextran-488 (10,000 MW); scale bar = 5 μm. (B and C) Vero cells pretreated macropinocytosis activation inhibitor (EIPA, 25μM) and incubated with PEAV (MOI = 20, 30 min); red: anti-PEAV N, green: anti-TSG101, blue: anti-DAPI; scale bar = 5 μm. More than 60 cells were included in the quantitative fluorescence density analysis. (D and E) Vero cells or primary porcine jejunum cells (IEC) were pretreated with EIPA (15 min, 37°C), followed by inoculation with PEAV (MOI = 0.5) (1 h, 37°C) in the presence of drugs and further incubated in a drug maintenance medium (12 h, 37°C), followed by immunoblotting to detect the expression of PEAV or PEDV N protein. All results are presented as the mean ± SD from three independent experiments (**, *P* < 0.01).

Recent studies have shown that TSG101 is associated with KSHV macropinocytosis and genomic nuclear transmission [20]. Meanwhile, we reported that PEAV primarily enters host cells by inducing macropinocytosis [21]. Therefore, we evaluated whether the TSG101-dependent difference in PEDV and PEAV entry into cells was related to macropinocytosis activation. To this end, we observed the co-localization of TSG101 PEAV or PEDV with Dextran—a macropinocytosis marker. The results showed that 30 min after infection, PEAV and Dextran were significantly co-localized with each other and with TSG101. However, no obvious co-localization was observed between PEDV, Dextran, and TSG101 (Fig 4A). In another approach, we analyzed the ability of PEDV/PEAV to induce membrane ruffling, actin remodeling, or an increase in dextran uptake. To this purpose, Vero cells were infected with PEDV/PEAV at 37°C for 15 min. As a positive control, cells were either treated with phorbol esther PMA. The results showed that, no significant induction of membrane ruffling, or actin reorganization was observed in PEDV-infected cells. At PMA or PEAV induced extensive actin reorganization (S3A Fig). Consistent with these results, PEDV infection did not no significant increase whereas PMA treatment or PEAV infection increased dextran uptake (S3B and S3C Fig).

To further determine whether the co-localization of TSG101 and PEAV depends on macropinocytosis activation, we treated Vero cells with a macropinocytosis activation inhibitor, EIPA. The results showed that the co-localization ratio of TSG101 and PEAV decreased significantly after EIPA treatment (Fig 4B and 4C). Consistently, EIPA dose-dependently inhibited the expression of the PEAV N protein in Vero and IEC, while showing no significant inhibitory effect on the expression of the PEDV N protein (Fig 4D and 4E). Hence, these results suggest that the difference in TSG101 dependence between PEDV and PEAV during the entry process might be caused by different degrees of viral dependence on macropinocytosis.

### ALIX and TSG101 are required for PEDV/PEAV infection in primary jejunal epithelial cells

**Fig 5:**
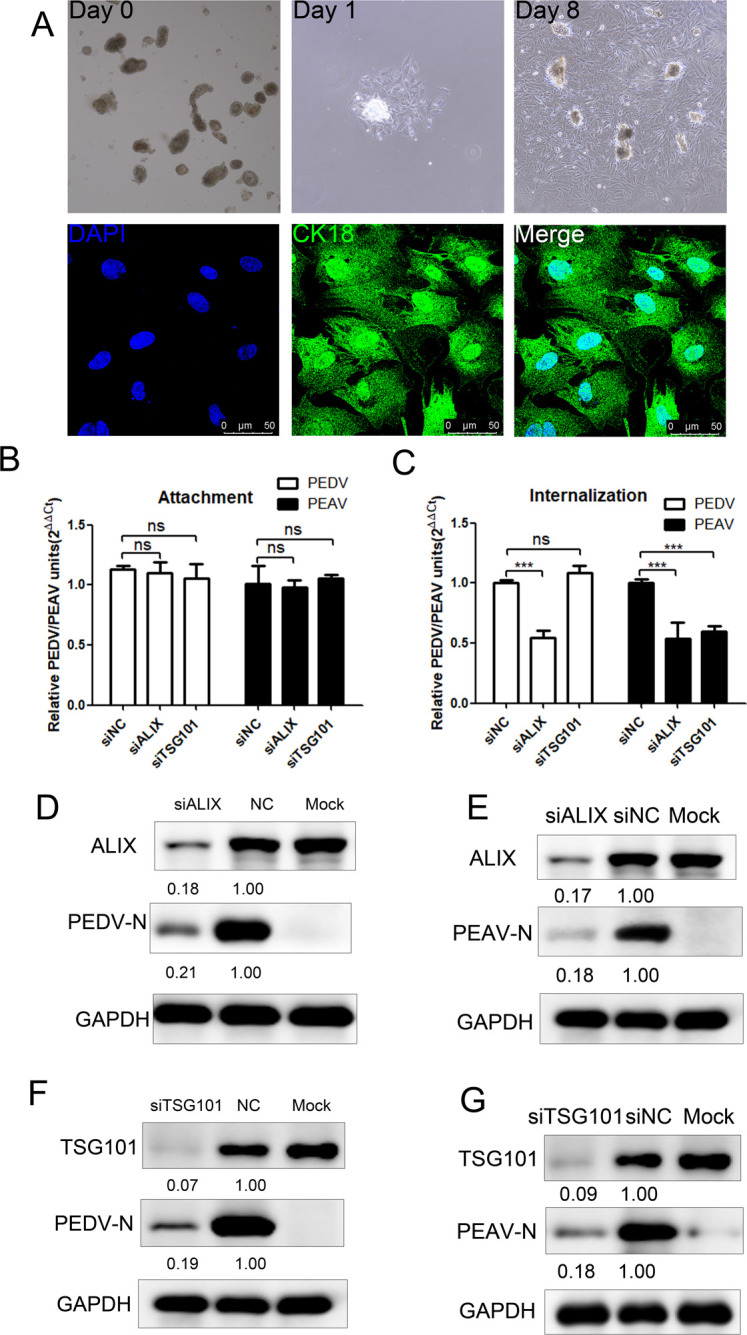
ALIX and TSG101 are required for PEDV and PEAV to infect primary pig jejunum. (A) Morphology of primary porcine jejunum villi cultured after 0–8 dpi (10*×*magnification); scale bar = 200 μm. Epithelial cell surface marker Cytokeratin 18 was used to determine cell purity; scale bar = 50 μm. (B and C) (B) Attachment (1 h,4°C), (C) internalization (1 h,37°C) of PEDV or PEAV by Vero cells transfected with siALIX, siTSG101 or siCtrl for 48 h before PEDV or PEAV infection. Protease K (0.5 mg/ml) for 5 min at 4°C to remove adsorbed but not internalized virus. (D to G) Vero cells transfected with (D and E) siALIX, (F and G) siTSG101, or siCtrl for 48 h, and infected with PEDV or PEAV (MOI = 0.1, 48 h); (D and E) anti-ALIX, (F and G) anti-TSG101, (D and F) anti-PEDV N, (E and G) anti-PEAV N. GAPDH is the loading control. All results are presented as the mean ± SD from three independent experiments (***, *P* < 0.001; ns, P > 0.05).

Although PEDV and PEAV both required ALIX and TSG101 for replication in immortalized Vero cells, we wanted to ensure that any differences in virus-dependent host factors were not caused by metabolic differences in immortalized cells and, thus, performed similar studies in jejunal epithelial cells from newborn piglets to investigate whether the two coronaviruses also require ALIX and TSG101 in primary cell lines. The intestinal epithelial cell marker, CK18, was used to confirm cell morphology and purity (Fig 5A). Next, we assessed the effect of ALIX or TSG101 depletion on viral entry. Compared to the control siRNA treatment, the depletion of ALIX or TSG101 did not impact the adsorption of PEDV and PEAV (Fig 5B). However, the depletion of ALIX significantly diminished the internalization of PEDV and PEAV, while the depletion of TSG101 had no effect on PEDV internalization but notably reduced PEAV internalization (Fig 5C). To evaluate the roles of ALIX and TSG101 in PEDV or PEAV infection, we depleted ALIX and TSG101 expression in primary jejunal epithelial cells using ALIX- and TSG101-specific siRNAs. Following ALIX (Fig 5D and 5E) or TSG101 (Fig 5F and 5G) depletion, PEDV and PEAV N protein expression was significantly reduced. This is consistent with Vero cell results, indicating that the replication process of PEDV and PEAV requires ALIX and TSG101, which may be characteristic of alphacoronaviruses and independent of the cell line.

### ALIX and TSG101 are required for PEDV/PEAV replication

**Fig 6:**
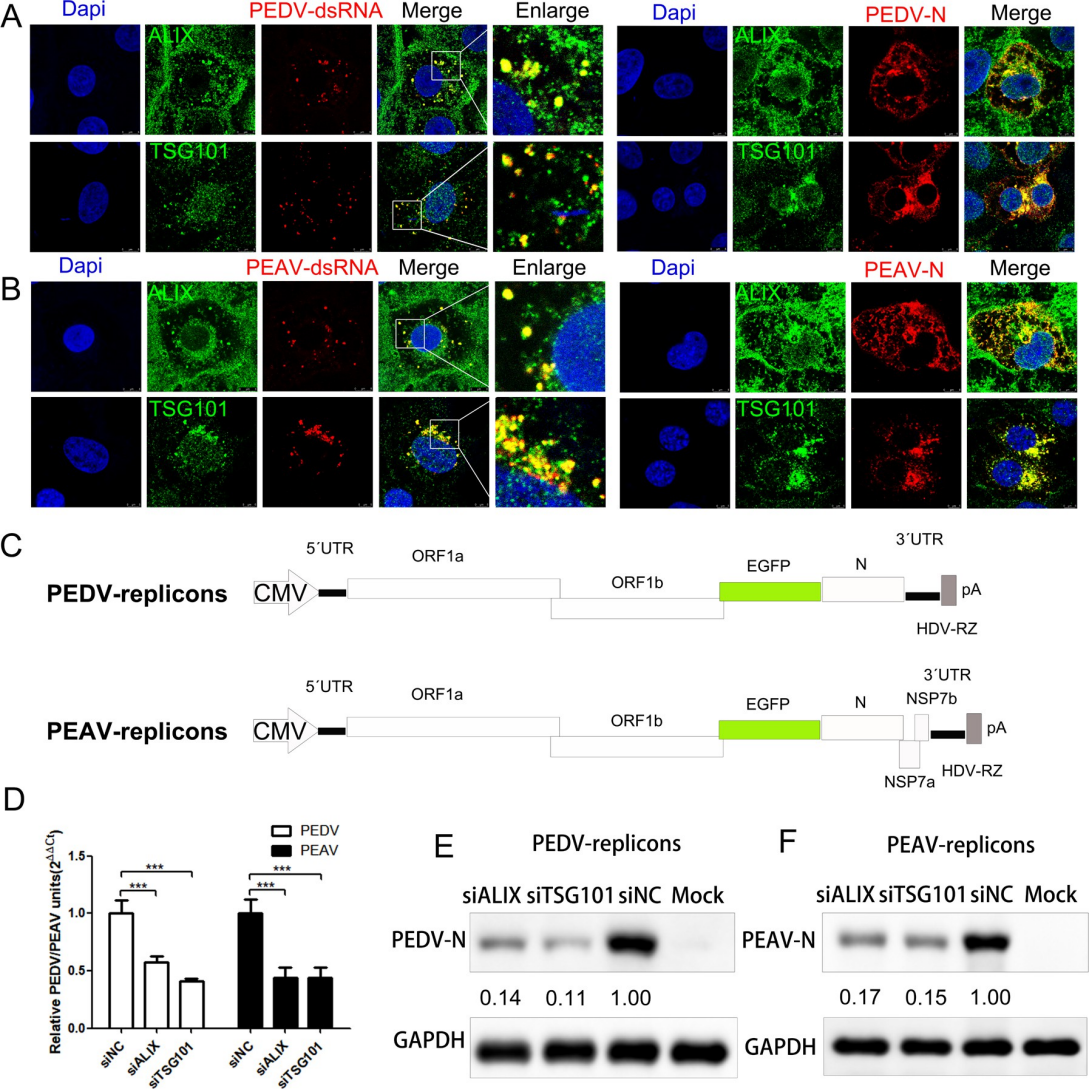
ALIX and TSG101 participate in PEDV and PEAV replication. (A and B) Vero cells infected with PEDV (A) or PEAV (B) (MOI = 0.5, 12 h); red: anti-dsRNA or anti-PEDV N or anti-PEAV N, green: anti-ALIX or anti-TSG101; Scale bar = 5 or 8 μm. (C) Schematic representation of PEDV or PEAV replicon system. CMV promoter (CMV), hepatitis delta virus ribozymes (HDV-RZ) and polyadenylation signal (pA). The fragments were assembled and sequentially cloned into a modified BAC plasmid. (D to F) PEDV or PEAV RNA levels in Vero cells transfected with siALIX, siTSG101, or siCtrl. (E) PEDV, (F) PEAV, and PEDV or PEAV N protein abundance in Vero cells transfected with siALIX, siTSG101, or siCtrl (48 h) and transfected with replicon of PEDV or PEAV (5 μg, 24 h). GAPDH is an internal control (***, *P* < 0.001).

To determine whether ALIX or TSG101 are involved in viral replication, we infected Vero cells with PEDV or PEAV and observed the localization of ALIX or TSG101 with viral dsRNA using confocal microscopy. As expected, the dsRNA or N protein induced by PEDV (Fig 6A) or PEAV (Fig 6B) infection was significantly co-localized with endogenous ALIX and TSG101. This suggests that although the entry process of PEDV does not require TSG101, both ALIX and TSG101 have important roles in PEDV and PEAV replication. To measure the replication of PEDV or PEAV, we developed a recombinant replicon system via reverse genetics by replacing S gene region with EGFP. Furthermore, we deleted the structural protein genes and retained the N gene and essential promoter regions (Fig 6C). In these experiments, we depleted ALIX or TSG101 in Vero cells and transfected cells with viral replicon. Compared with the control siRNA treatment, ALIX or TSG101 knockdown significantly decreased viral RNA levels in cells (Fig 6D). Similarly, following ALIX or TSG101 depletion, PEDV (Fig 6E) and PEAV N (Fig 6F) protein expression was significantly reduced.

### ALIX and TSG101 participates in the formation of PEDV/PEAV replication complex

**Fig 7:**
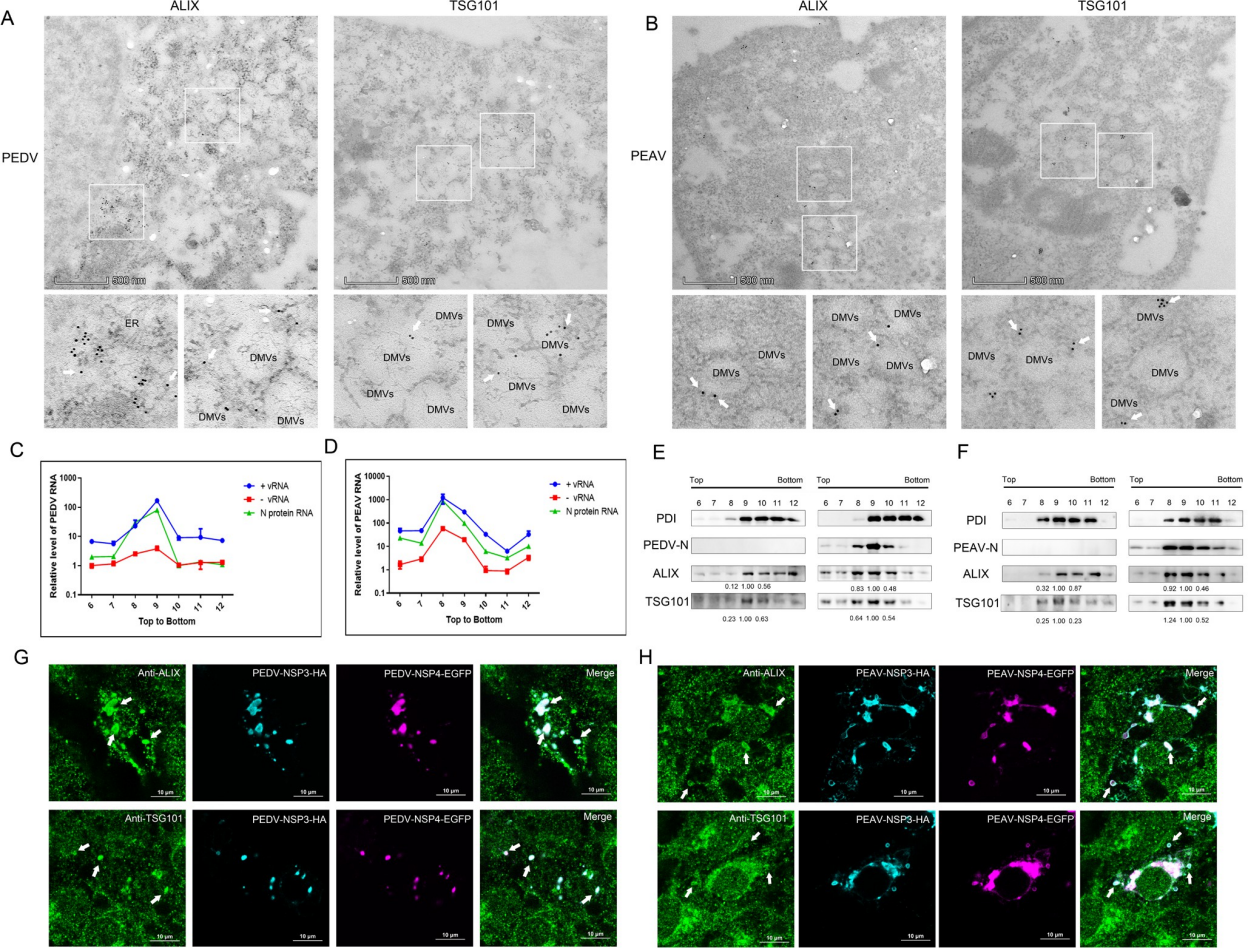
ALIX and TSG101 localized within the endoplasmic reticulum or virus-induced double-membrane vesicles. (A and B) Vero cells infected with (A) PEDV or (B) PEAV and treated with anti-ALIX or anti-TSG101 (white arrow) (MOI = 0.1, 24 h). Cells observed using FEI/Talos L120C transmission electron microscopy. Scale bar = 5 μm. ER: endoplasmic reticulum; DMVs: double-membrane vesicles. (C and D) Vero cells infected with PEDV or PEAV (MOI = 0.1) and harvested at 24 h. The cells are lysed with homogenizer, and then centrifuged (2,500×g, 10 min, 4°C) to pellet cellular debris and nuclei. The supernatant (containing 5 mg protein) was divided into 12 fractions through membrane flotation assay. Collect 6–12 fractions of membrane flotation assay (1 mL) each from top to bottom, and 200 μL fractions were harvested for total RNA extraction (MOI = 0.1, 24 h). Relative amounts of (C) PEDV or (D) PEAV positive- and negative-strand RNA or N protein mRNA were quantified using RT-qPCR. GAPDH is the internal control. (E and F) Same as (C and D) (E) PEDV or (F) PEAV, and take different layer fractions for SDS-PAGE and Western blots to determine the abundance of endoplasmic reticulum marker proteins: PDI (Proteintech, USA), PEDV or PEAV N protein, endogenous ALIX, and TSG101 after infection. (G and H) Vero cells transfected with PEDV/PEAV-NSP3-HA, PEDV/PEAV-NSP4-EGFP plasmids for 24 h. Immunostaining reveals that ALIX or TSG101, PEDV/PEAV-NSP3-HA, PEDV/PEAV-NSP4-EGFP, green: anti-ALIX or TSG101, cyan: anti-HA, light purple: EGFP, and endogenous ALIX or TSG101 accumulate at the NSP3-HA/NSP4-EGFP+ punctate structures, as indicated by arrows. Scale bar = 10 μm.

Many studies have also reported on the formation of viral replication complexes (VRC) by ESCRT, such as classical swine fever virus (CSFV) [22] and brome mosaic virus (BMV) [23]. As alphacoronavirus rearrange the ER of host cells to form DMVs, a platforms for viral replication [12], we thus speculated that ALIX and TSG101 may be recruited to DMV to promote virus replication. To test this hypothesis, we analyzed the distribution of ALIX and TSG101 by transmission electron microscopy (TEM) in PEDV or PEAV-infected cells. As expected, we observed the localization of ALIX and TSG101 in the endoplasmic reticulum or double-membrane vesicle structure after PEDV and PEAV infection (Fig 7A and 7B). Next, we performed membrane flotation assays to extract and purify VRC from the Vero cells infected with PEDV or PEAV. We used strand-specific RT-qPCR to distinguish the production of positive-strand and negative-strand viral RNA (+vRNA and -vRNA), and the enrichment layer of positive and negative stranded viral RNA represents the enrichment of VRC. As shown in Fig 7C and 7D, the VRC of PEDV and PEAV were enriched in the 9th and 8th layers, respectively. Consistently, the protein levels of ALIX and TSG101 in the VRC were significantly increased by the viral infection (Fig 7E and 7F). Previous studies revealed that the coexpression of coronavirus NSP3 and NSP4 induces the formation of zippered ER and DMVs [24,25]. We transiently transfected PEDV or PEAV NSP3 and NSP4 and found that the coexpression of both proteins led to the formation of a large number of discrete puncta or vesicular structures, which were co-localized with endogenous ALIX and TSG101 (Fig 7G and 7H). These results indicate that ALIX and TSG101 are recruited to the viral genome replication site during viral infection, and play important roles in the PEDV/PEAV DMVs formation and replication.

## Discussion

**Fig 8:**
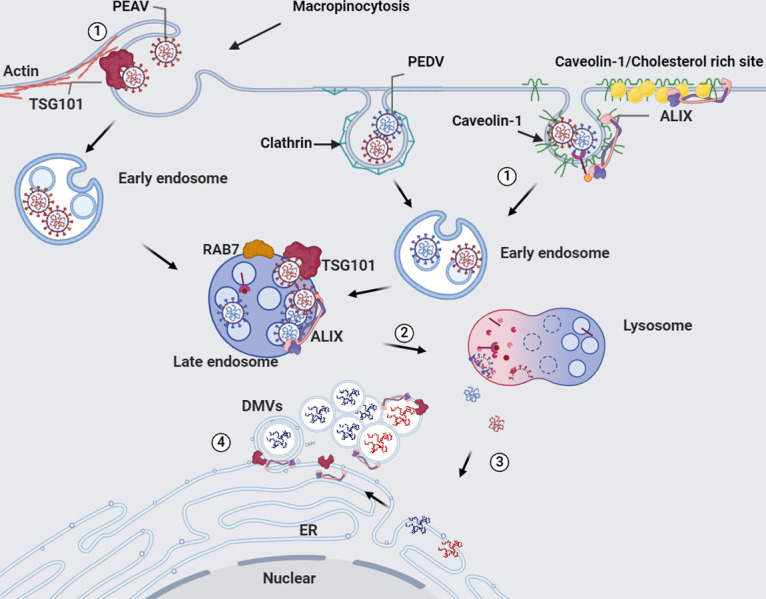
Schematic model depicting the life cycle of two porcine alphacoronaviruses and the potential roles of ALIX and TSG101. (1) PEDV/PEAV induce endocytosis by binding to cell receptors, where ALIX can be recruited to the CAV1 rich site, and TSG101 can be recruited to macropinocytosis endocytosis site in PEAV, followed by virus internalization. (2) Subsequently, the virus is transported from early endosomes to lysosomes and ALIX and TSG101 are recruited to late endosomes, promoting viral transport to lysosomes. (3) In a low pH environment, the virus capsid disassembles and disrupts the outer viral membrane, releasing the genome into the endoplasmic reticulum. (4) Ultimately, ALIX and TSG101 are recruited into the endoplasmic reticulum to help PEDV/PEAV form double membrane vesicles and promote viral replication.

In this study, we analyzed the dual role of ALIX and TSG101 in the entry and replication of alphacoronaviruses. The results summarized in Fig 8 indicate that ALIX is recruited to CAV1-dependent endocytic vesicles, promoting the internalization of PEDV and PEAV. Following endocytosis, the virus enters late endosomes (expressing RAB7) and continues to recruit ALIX to promote transport from the endosomes to lysosomes, thus, completing the viral genome release. Meanwhile, TSG101 is recruited to macropinocytosis-dependent endocytic vesicles to regulate PEAV internalization; TSG101 is also recruited after endocytosis to promote transport from endosomes to lysosomes and to complete the release of the viral genome. In addition, both PEDV and PEAV replication processes require ALIX and TSG101, which are located in DMVs. Hence, we have identified the roles of ALIX and TSG101 in the entry and replication of PEDV and PEAV. That is, ALIX and TSG101 are recruited to endocytic vesicles, participate in virus internalization and intracellular transport, and are recruited to viral replication sites to support the viral replication process.

Viruses often hijack basic and conserved host cellular pathways or mechanisms by utilizing host cell resources to produce new viral particles. The host endosomal sorting complex required for the transport system (ESCRT) is a conserved molecular machine in eukaryotic cells that participates in important life processes, including cell cytokinesis, endosomal maturation, autophagy, membrane repair, and reconstruction [9]. Among these, ESCRT mediates the formation of MVB and autophagosomes, similar to the formation of replication organelles (ROs) and viral budding in topology. Many studies have shown that ESCRT is involved in the budding process of various viruses, such as the feline immunodeficiency virus (FIV) [26], Sendai virus (SEV) [27], and human immunodeficiency virus (HIV) [17]. In addition, the process of MVB formation is crucial for endosome maturation. In fact, the Crimean-Congo hemorrhagic fever virus relies on ESCRT components to enter cells via MVB [28]. Meanwhile, TSG101, VPS25, CHMP4B, and CHMP7 play important roles in clathrin-mediated endocytosis of classical swine fever virus [22,29]. Hence, it follows that ESCRT is also involved in viral entry.

Considering that replication of SARS-CoV-2 in severe cases requires a large number of ESCRT complexes, other coronaviruses might also require an ESCRT system [30]. However, to the best of our knowledge, the specific role of ALIX or TSG101 in the entry and replication processes of alphacoronaviruses has not been previously reported. Our study found that within 30 min of infection with PEDV or PEAV, ALIX was enriched in CAV1 rather than Clathrin or RAB5. Clathrin-mediated endocytosis often recruits ESCRT [31]. For instance, CSFV infection promotes the co-localization of multiple ESCRT subunits with Clathrin [22]. Early studies have shown that ubiquitin-labeled membrane proteins are typically transported to endosomes with the assistance of ESCRT, many of which are membrane-bound receptors that ultimately target lysosomal degradation [32–34]. Therefore, we speculate that the increased interaction between ALIX and CAV1 may represent signal transduction activated by viral binding to cells. This signal transduction recruits ALIX to CAV1 rich regions, such as cholesterol-rich regions, thereby initiating viral endocytosis. Considering that recent studies have shown that PEDV and PEAV depend on caveolae- and clathrin-mediated endocytosis [19,21], we postulate that the virus directly recruits ALIX, resulting in a significant increase in the interaction of ALIX and Clathrin or CAV1. Our results showed that there was no significant interaction between ALIX and viral structural proteins (S4A and S4B Fig). That is, the increased interaction between ALIX and CAV1 induced by PEDV and PEAV infection is likely due to the activation of signal transduction pathways following virus binding, subsequently recruiting ALIX to CAV1-rich regions, rather than by direct recruitment through virus surface structural proteins. This is similar to KSHV, which recruits TSG101 to regulate viral entry by binding to host cells to induce a signaling cascade reaction [20,35].

Moreover, we observed ALIX was enriched in RAB7 following PEDV and PEAV internalization. However, in ALIX-depleted cells, the virus accumulated in endosomes without complete genome release. Hence, ALIX also has an important role in the late endosomal-to-lysosomal transport of the virus. Early studies reported that in mammalian cells, depletion of ALIX leads to a 50% reduction in unconventional phospholipid hemolytic double phosphatidic acid (LBPA) in MVB. LBPA has the ability to drive acidic lipids into endosomes, thereby promoting endosomal acidification [36,37]. Therefore, depletion of ALIX may lead to inhibition of endosome acidification. Consequently, the acidity of the endosome is insufficient to trigger the fusion of the viral envelope and endosomes, and the genome release process will be inhibited. This suggests that the unique demand for ALIX during viral endocytosis and late endosome-to-lysosomal transport may be a common feature among alphacoronavirus infection and yet to be characterized.

TSG101, as a core component of ESCRT-I, plays an important role in MVB biogenesis, and supports the efficient budding of several enveloped viruses [38–40]. Our results indicate that PEDV entry into Vero cells does not require TSG101, whereas PEAV entry does. Initially, we posited that PEDV might interact with ALIX through viral proteins and that ALIX directly interacts with CHMP4B through its Bro1 domain, thus directly forming ESCRT-III and recruiting the ESCRT system to complete the virus infection cycle [7]. In this way, PEDV would not require TSG101 to recruit the ESCRT system. However, no significant interactions were observed between PEDV and PEAV structural proteins or ALIX. Therefore, we speculated that the PEAV M protein contains a key proline conserved motif “PVAP” (similar to the TSG101 recruitment motif, whereas PEDV does not have a similar motif. However, the mutated PEAV M protein motif “AVAA or PVAA” interacted with TSG101 (S5A and S5B Fig). Additionally, the PEDV M protein can interact with TSG101 (S5C Fig). Moreover, as the PEDV or PEAV M protein is continuously truncated, the interaction region was identified as 156–172aa, which did not contain similar late structural motifs but contains a proline conserved region "PXFV" (S5D–S5H Fig), suggesting that PEDV and PEAV may recruit TSG101 to participate in viral infection through other conserved motifs or in more complex ways, which requires further investigation. However, this result indicates that PEAV entry depend on TSG101 is not due to the interaction between viral structural proteins and TSG101.

Previous studies showed that TSG101 is involved in clathrin-mediated endocytosis [31], while our recent report showed that PEAV entry requires clathrin-mediated endocytosis [21]. Therefore, we speculate that PEAV entry requires TSG101 and clathrin-mediated endocytosis. However, our results indicated that PEDV or PEAV infection did not increase the the interaction between TSG101 and clathrin. Hence, other PEAV endocytic pathways may require TSG101. Meanwhile, KSHV was found to require TSG101 to enter endothelial cells through macropinocytosis [20]. Interestingly, PEAV entry more depends on the macropinocytosis pathway to enter cells, which may be the main endocytic pathway [21]. Therefore, we speculated that the dependence of PEAV on TSG101 may be related to the activation of macropinocytosis. Subsequent experiments demonstrated that PEAV infection for 30 min could induce co-localization of TSG101 and dextran—macropinocytosis marker. Whereas, treatment with EIPA—a macropinocytosis inhibitor—significantly inhibited TSG101 and PEAV colocalization. Contrary to PEAV, our results do not support PEDV activation of macropinocytosis since classical cues of triggered macropinocytosis such as the induction of membrane ruffling, or the increase of dextran uptake, were not detected. In addition, PEAV infection increased the interaction between TSG101 and RAB7. This suggests that TSG101 plays an important role in the PEAV macropinocytosis pathway and lysosomal transport. This was further confirmed by the accumulation of viruses in the endosomes of TSG101-depleted cells. Indeed, TSG101 depletion reportedly directly inhibits MVB formation and endosomal maturation [41]. Therefore, TSG101 and ALIX may affect the late endosome-to-lysosomal transport of the virus by inhibiting endosomal acidification or maturation.

Although there was a difference in the requirement for TSG101 between PEDV and PEAV, ALIX and TSG101 depletion significantly inhibited PEDV and PEAV replication. ESCRT has the potential to induce the formation of viral replication organelles [9]. Hence, the alphacoronavirus may have evolved the ability to induce the formation of viral replication organelles through ESCRT. Depending on the virus family, different host membrane structures are used to produce replication organelles, including the endoplasmic reticulum, peroxisomes, Golgi bodies, and endosomes [42]. For example, tombusvirus (TBSV) replicates in peroxisomes, a process that is ESCRT-dependent [43]. Meanwhile, CSFV hijacks ESCRT components, such as HRS, ALIX, and VPS4A to regulate the formation of replication organelles in the endoplasmic reticulum [22]. As expected, endogenous ALIX and TSG101 significantly colocalized with the viral replication marker dsRNA and viral N protein in PEDV- and PEAV-infected cells, indicating that ALIX and TSG101 play an important role in viral replication. Double-membrane vesicles (DMVs) are generally considered viral replication organelles induced by coronaviruses in the endoplasmic reticulum [12,44]. We observed the localization of ALIX and TSG101 in the endoplasmic reticulum or double-membrane vesicle structure after PEDV and PEAV infection, indicating that ALIX and TSG101 may have important roles in the formation of alpha-coronavirus DMVs. However, Japanese encephalitis virus (JEV) and dengue virus (DENV) rely on ESCRT to form viral particles rather than replication organelles [14]. Similar results have been reported for hepatitis C virus (HCV) [45] and porcine reproductive and respiratory syndrome virus (PRRSV) [46]. This indicates that the regulation of virus replication by ALIX and TSG101 is not only related to DMV formation but may also be associated with the formation of mature viral particles. However, it is difficult to determine whether ESCRT participates in DMV formation, virion assembly, or both. For example, the absence of ESCRT components did not lead to changes in the morphology of JEV-induced ROs; however, the ESCRT protein was involved in JEV infection-induced endoplasmic reticulum membrane deformation and was located in the ROs [14]. Therefore, it will be challenging to separate the study of ESCRT-mediated viral ROs from the mature viral particle formation processes.

This study used membrane flotation experiments to roughly separate the replicating organelles of coronavirus, and found that ALIX and TSG101 were enriched together with the endoplasmic reticulum, which also suggests that they may play important roles in endoplasmic reticulum rearrangement. However, ALIX and TSG101 migrate as the abundance of PEDV or PEAV ROs increases, indicating that viral replication may require recruiting ALIX and TSG101 to DMVs. Subsequently, we induced the formation of minimal units of DMVs using NSP3 and NSP4, and found that endogenous ALIX and TSG101 exhibited puncta or vesicular aggregation with DMVs. These results suggest that ALIX and TSG101 play an important role in the formation of DMVs in PEDV and PEAV.

## Conclusions

This study presents new roles for ALIX and TSG101 in the entry and replication mechanisms of PEDV and PEAV. Both PEDV and PEAV exhibited similar results in terms of the demand for ALIX and TSG101 in susceptible Vero and primary jejunal epithelial cells. Hence, the elucidated properties could be shared by other numerous species of alphacoronaviruses that infect non-porcine hosts and yet to be characterized. However, due to the unknown PEDV or PEAV receptor, this study had certain limitations and cannot determine the direct signal of ESCRT recruitment during the coronavirus entry process based on the receptor. In addition, whether ESCRT directly regulates the formation of DMVs or indirectly regulates it through other molecules remains unresolved. As such, further biochemical analyses of ALIX and TSG101, as well as other components of the ESCRT system, may identify additional therapeutic targets for novel antiviral drugs against coronavirus infection.

## Materials and methods

### Ethics statement

All experiments described in this study were reviewed and approved by the Experimental Animal Ethics Committee of South China Agricultural University. The study was conducted in accordance with the local legislation and institutional requirements.

### Cell culture

Vero and 293T cells were preserved in our laboratory and maintained in Dulbecco’s modified Eagle’s medium (DMEM) containing 10% fetal bovine serum (FBS; Gibco). Primary porcine small intestinal epithelial cells were obtained from the jejunal villi of 0-day-old piglets, maintained in 1*×*F12/DMEM medium (Gibco) with 5% FBS, 15 ng/mL EGF, and ITS (insulin, transferrin, and selenium) additive (Sigma), all cells were maintained at 37°C in a humidified atmosphere of 5% CO_2_.

### Antibodies and reagents

The sources of the antibodies for the different markers were as follows: ALIX (rabbit polyclonal) and TSG101 (rabbit polyclonal) for immunoprecipitations and Immunofluorescence, ALIX (mouse mAb, clone 1H9D9) and TSG101 (mouse mAb, clone 2B7G8) for Western blotting, GAPDH (mouse mAb, clone 1E6D9), Clathrin (mouse mAb; clone 1B3D7), RAB5 (mouse mAb, clone 1B6A5), RAB7 (rabbit polyclonal), Cytokeratin 18 (rabbit polyclonal) and PDI (rabbit polyclonal) were purchased from Proteintech. Caveolin-1 (rabbit polyclonal), Caveolin-1 (mouse mAb, clone 7C8) and RAB5 (rabbit polyclonal) were purchased from Abcam. dsRNA (mouse mAb, clone J2) was from Scicons. HA (mouse mAb, clone 2–2.2.14) was from Invitrogen. Flag (mouse mAb, clone M2) was from Sigma. Mouse monoclonal anti-PEDV/PEAV N antibodies were generated in our laboratory.

Secondary antibodies conjugated to Alexa-488, -594, or -647 were from Proteintech. Goat Anti-Rabbit/Mouse IgG H&L (HRP) were from Abcam. Alexa Fluor 647 NHS Succinimidyl Esters (A37573) and Alexa Fluor 488-labeled dextran (D22910) were from Invitrogen. Actin-Tracker Green-488 was from Beyotime. Protease K and EIPA (S9849) were purchased from Sigma. PMA (S7791) was from Selleck.

### Viral stock production and titration assay

All viruses were produced in Vero cells. Briefly, Vero cells were cultured into a 100 mm cell culture dish and grown to 100% confluence; they were then washed three times with phosphate-buffered saline (PBS), inoculated with PEDV at an MOI of 0.1, and incubated in DMEM with 10 μg/mL trypsin at 37°C for 48 h. The culture was collected and freeze-thawed three times, centrifuged at 10,000*×g* for 10 min, and the supernatant was collected and stored at -80°C.

Virus titers were determined by seeding Vero cells into 96 well plates, and at 100% confluence at a density of 10^5^ cells per well. Subsequently, the virus solution was diluted along a ten-fold gradient using DMEM containing 10 μg/mL trypsin (virus maintenance medium). Cells were pre-washed three times with PBS and inoculated with 100 μL of virus solution at different dilutions. Each concentration was added to eight wells. The cells were cultured in virus maintenance medium for 48 h and cytopathic effects were recorded. The virus titer was calculated using the Reed Munch method.

### Plasmids and siRNA

All plasmids were constructed using the pCAGGS-HA vector. The reference sequence for the PEAV structural protein and its mutant was the GDS04 strain (Access: MF167434.1); the reference sequence for the PEDV structural protein and its mutant was the GDS01 strain (Access: KM089829.1); the reference sequences for ALIX and TSG101 were Access: XM_ 008009430.2 and Access: XM_ 008004714.2. Using Vero cells or viral cDNA as a template, the target gene fragment was obtained by PCR amplification, and the PEAV M protein motif mutant gene fragment was obtained by the fusion PCR method. The recombinant plasmids were confirmed by sequencing. The reference sequences for the ALIX and TSG101 siRNAs from green monkeys were the same as those of the plasmids, while the reference sequences for ALIX and TSG101 siRNAs from pigs were Access: XM_ 021071622.1, and Access: JN882576.1. All siRNAs were purchased from RiboBio (Guangzhou, China). All primers and siRNA-targeting gene sequences are presented in S1 and S2 Tables.

### Transfection of plasmids and siRNA

Vero cells were seeded into a 6-well plate and grown to 70–80% confluence. Lipofectamine 3000 Reagent was then added with 2.5 μg of plasmid per well for 24 h. An empty plasmid was used as the control.

293T cells were seeded into a 100-mm cell culture dish and grown until 60–70% confluence. Plasmid transfection was performed using PEI (FUSHENBIO). ALIX and TSG101 plasmids were co-transfected with 5 μg of structural protein or empty plasmids for 36 h.

Vero cells or porcine primary small intestinal epithelial cells were seeded into a 6-well plate and grown until 50–60% confluence. Lipofectamine RNAiMAX Reagent (Invitrogen) was added with 25 nM siRNA per well for 48 h. Negative control siRNA was used as a control.

### Virus purification and fluorescence labeling

The virus supernatant was centrifuged at 100,000*×g* and 4°C for 1.5 h. The supernatant was discarded, and the virus was resuspended in PBS. The virus suspension was added to a sucrose gradient (10–60%) and centrifuged for 2 h (4°C, 120,000*×g*). Subsequently, 50–60% of the layers were collected, PBS was added and the solution was centrifuged for 2 h (4°C, 40,000*×g*) to remove sucrose. Virus was then resuspended with PBS to obtain purified virus particles. Purified virions were covalently labeled with Alexa Fluor 647 NHS Ester fluorescent labeling dye at a final concentration of 5 μg/mL. After incubation at room temperature for 1 h, virions were centrifuged through a 10 KDa ultrafiltration tube for 15 min (4°C, 5,000*×g*), and PBS was added for repeated centrifugation three times to remove excess stain. Purified viruses and fluorescently-labeled viruses were collected and stored at -80°C.

### Construction of replicons

Replicon were produced as described previously [47,48]. Briefly, the genome was divided into seven fragments and subcloned into seven separate plasmids. A CMV promoter was introduced into the 5’ end, and four of the viral proteins (S, ORF3a, E, M) were replaced with EGFP. At the 3’ end, the replicon was assembled with a 25 bp poly(A) tail (pA25), followed by a hepatitis delta virus (HDV) ribozyme (Rz) and a bovine growth hormone (BGH) termination to generate replicon RNAs bearing authentic 3’ ends. Unique type IIS restriction endonuclease cleavage sites (BsaI-HF v2, NEB #R3733) are introduced into the 5’ and 3’ ends of each fragment. Plasmid DNA was digested with the corresponding enzymes, gel purified, and ligated together with T4 DNA ligase (NEB). Ligation products were cloned into the BAC vector and transformed into chemically competent HB101 cells. After determination of all the fragments by PCR, and the correct cloned plasmid is used for subsequent replicon detection experiments after sequencing.

### VLPs production

Virus-like particles (VLPs) were produced as described previously [49,50]. Briefly, equimolar amounts of full-length CoV S, E (envelope), M (membrane), and N (nucleocapsid) (total, 20 μg) were transfected into 10^7^ HEK293T cells. At 6 h post-transfection, cells were replenished with fresh DMEM-10% FBS. VLP suspensions were collected in FBS-free DMEM from 24 to 48 h post-transfection. Suspensions containing VLPs were clarified by centrifugation (300 g, 4°C, 10 min; 3,000 g, 4°C, 10 min). To obtain purified viral particles, clarified VLP suspensions were concentrated 100-fold by overlaid onto 20%, wt/wt, sucrose cushions and particles purified via slow-speed pelleting (SW28, 6500 rpm, 4°C, 24 h or SW32 8000 rpm, 4°C, 24 h). The resulting pellet was resuspended in FBS-free DMEM or HBSS to 1/100 of the original medium volumes. VLPs were stored at -80°C or analyzed promptly.

### Co-immunoprecipitation (Co-IP) and western blotting

Cells were lysed in radioimmunoprecipitation assay (RIPA) lysis buffer for 30 min at 4°C. Cell lysates were collected by centrifugation at 10,000*×g* and 4°C for 10 min, and proteins in the lysates were separated using SDS-PAGE. Proteins were transferred onto polyvinylidene fluoride (PVDF) membranes (0.45-μm pore; Merck Millipore) and probed with the indicated antibodies (mouse anti-ALIX, TSG101, Clathrin, RAB5, and rabbit anti-RAB7 antibody were purchased from Proteintech). GAPDH (Proteintech, USA) was used as a loading control. For immunoprecipitations, the 20% aliquot of the supernatant (whole cell lysate) was removed from all samples for later use. The 80% remaining lysate was incubated with 0.5 μg of the appropriate control IgG and 20 μL of a Protein A/G PLUS-Agarose slurry (Santa-cruz, USA) for 4 h at 4°C with rotation. Agarose beads were removed by centrifugation at 1,000×g for 5 minutes at 4°C. The lysates were then incubated with rabbit anti-ALIX or TSG101 antibody (Proteintech, USA). Protein A/G PLUS-Agarose slurry was added to each sample and incubated continued for another 2 h under the same conditions. The agarose beads were collected by centrifugation and washed with NP-40 lysis buffer at least three times, then resuspended in 2*×*SDS loading buffer for SDS-PAGE and Western blotting. For Co-immunoprecipitations, cells were lysed for 30 min at 4°C in IP buffer (Beyotime). Cell lysates were collected by centrifugation at 10,000*×g* and 4°C for 10 min, and supernatants were incubated with specified Flag antibodies conjugated to magnetic beads for 16 h at 4°C. Subsequently, the magnetic beads were washed with 500 μL PBST (0.5% Tween-20), collected, added to SDS-PAGE protein loading buffer, and heated at 95°C for 5 min. The supernatant was then collected for western blotting and the magnetic beads were discarded.

### Primary porcine enterocyte isolation

All animal experiments were approved by the Animal Care and Use Committee of the South China Agricultural University and followed the guidelines of the National Institutes of Health.

Porcine enterocytes were extracted and isolated from the jejunal tissues of 0-day-old piglets. Briefly, tissue sections were cut into 5 cm pieces, washed with ice-cold PBS, and placed in 50-mL conical tubes containing 1*×*antibiotic-antimycotic. The intestinal segment was cut lengthwise, followed by vigorous shaking to remove intestinal contents or fully differentiated enterocytes, and washed with ice-cold PBS. The cleaned intestinal segments were placed in dishes with ice-cold PBS, and the intestinal villi were removed with a cell scraper and transferred to a 50-mL centrifuge tube with ice-cold PBS containing 2% FBS. The tubes were centrifuged for 3 min at 500*×g*, washed five times with ice-cold PBS, and the cellular precipitates were collected. Freshly isolated jejunal epithelial cells were seeded at a density of 250,000 cells/well and maintained in Ham’s F12 nutrient medium containing murine epidermal growth factor (Sigma-Aldrich) to stimulate epithelial cell growth and differentiation. Differentiated cells were grown on collagen hydrogels for the viral infection experiments.

### Transmission electron microscopy and immuno-electron microscopy

Vero cells infected with PEAV or PEDV were fixed with 2.5% glutaraldehyde in phosphate buffer (0.1 M, pH 7.0). After two washes with PBS and one wash in ddH2O, cells were postfixed in 1% OsO4 for 4 h; cells were then washed with water and placed in chilled 2% aqueous uranyl acetate overnight at 4°C. After washing with ddH_2_O, the cells were dehydrated using a graded ethanol series and embedded in epoxy resin. Embedded samples were cut into ultrathin frozen sections of 80 nm using an ultramicro slicer (Leica EM FC7). Slices were fixed on a copper mesh and observed under a transmission electron microscope (FEI/Talos L120C).

For electron microscopy, the fixed sampling method was the same as that used for sample preparation with a transmission electron microscope. After washing with ddH_2_O, the cells were placed in 1 mL of 0.1 M glycine solution for 1 h at room temperature. Cells were then washed again with ddH_2_O, dehydrated using a graded ethanol series, and embedded in epoxy resin. Similar to the TEM, slicing was performed using a nickel mesh. Slices were incubated with rabbit anti-ALIX (Proteintech, USA) or TSG101 (Proteintech, USA) antibodies and 10 nm colloidal gold-conjugated goat anti-rabbit IgG, and subsequently stained with 1% lead citrate. Finally, the samples were observed using a transmission electron microscope (FEI; Talos L120C).

### Immunofluorescence confocal microscopy

Vero cells were cultured in a 14-mm glass bottom cell culture dish (Cellvis). After different experimental treatments, the cells were fixed with 4% paraformaldehyde (PFA) for 15 min, washed three times with PBS at 20°C, and infiltrated with 0.3% Triton X-100 for 5 min. Cells were stained overnight with specific antibodies at 4°C or for 2 h at 37°C. The cells were washed three times with PBS and incubated with the corresponding secondary antibody at 37°C for 1 h. Cell nuclei were stained with 1 mg/mL DAPI (Beyotime).

### Membrane flotation assay

Membrane Flotation Assay as previously described by Dorothee A Vogt et al [51] with minor modifications. Briefly, Cells from a 100-mm plates were harvested, washed twice with ice-cold PBS, and resuspended in 2 mL 4°C PBS/Sucrose (8.55 g sucrose in 100 ml of PBS, and protease inhibitor). The cells are lysed with 200 passages in a tight-fitting dounce homogenizer, while keeping the homogenizer on ice. The homogenates were centrifuged at 2,500×g for 10 min at 4°C to pellet cellular debris and nuclei. The supernatant (containing 5 mg protein) was diluted with PBS/Sucrose and add PBS/Sucrose to a total volume of 2 mL. Discontinuous iodixanol gradients were generated in SW41 tubes (Beckman Instruments) by overlaying the following iodixanol solutions all in homogenization buffer: 4 mL of iodixanol/supernatant mixture in 30%, 4 mL in 20% iodixanol (pre-chilled), 4 mL in 10% iodixanol (pre-chilled). The gradients were centrifuged at 209,000×g (35,000 rpm) overnight (16 h) at 4°C in an SW41T rotor. Subsequently, 12 fractions (1 mL each) were collected from the top. Fractions were boiled with SDS-PAGE sample buffer and subjected to immunoblotting analysis.

### Cell viability assay

Vero or jejunal epithelial cells were transfected with siRNA (25 nM) or treated with drug inhibitors (0–300 μM). According to the manufacturer’s instructions, the CCK-8 kit was used to determine cell viability. The absorbance values at 450 nm were measured to determine cell viability. An average of 4 to 8 readings were taken. Cell survival rate (%) = (optical density [OD] of experimental group/OD of the control group) 100%. The effect of inhibitors or siRNA cell viability is shown in (S6 Fig).

### Statistical analysis

All data are presented as mean ± SD. Student’s *t*-test was used to compare data from pairs of treated and untreated groups. A *P* < 0.05 was considered statistically significant. All statistical analyses and calculations were performed using the Prism 5 software (GraphPad Software Inc., La Jolla, CA, USA).

The underlying numerical data and statistical analysis graph for all figures are included in S1 Data.

## Supporting information

S1 Fig Role of ALIX and TSG101 in the entry of PEDV or PEAV VLP into Vero cells. (A and B) (A) PEDV VLPs, PEAV VLPs, all of which were produced from 293T cells, were independently purified by sucrose gradient centrifugation and 5 μg of purified VLPs were applied to each lane of SDS-PAGE; a: S: N: M: E molar ratio is 1:1:1:1; b: S: N: M: E molar ratio is 1:2:1:1. (B) Negative staining of PEDV VLP, PEAV VLP. (C) Vero cells inoculated with PEDV VLP or PEAV VLP for 30 min (10 μg); red: anti-PEDV N or anti-PEAV N, green: anti-ALIX or anti-TSG101, blue: Dapi; white arrows represent co- located clusters. Scale bar = 10 μm. (D) Co-localization of TSG101 with PEDV or PEAV N expressed as Pearson’s correlation coefficient, measured for individual cells. All results are presented as the mean ± SD from three independent experiments (***, P < 0.001).
(TIF)

S2 Fig Transmission electron microscopy analysis of the intracellular transport characteristics of PEDV or PEAV in Vero cells. (A) The red circle represents a complete vesicles containing viruses, recorded as “+”; Scale bar = 5 μm. (B) The red circle represents a complete vesicles containing viruses and lysosomal structures, recorded as “-”; Scale bar = 5 μm. (C) Same as (B). (D) Mock-infected group. The red circle represents that there are no vesicles containing viruses or viral mixed lysosomal structures. Brown irregular lines: lysosomal structures.
(TIF)

S3 Fig PEDV does not induce macropinocytosis in Vero cells. (A) Vero cells were inoculated with PEDV/PEAV (MOI = 20) (1 h, 4°C), washed, incubated (15 min, 37°C), and fixed (4% PFA, 15 min, RT). Vero cells were pretreated with PMA (positive control) (200 nM, 60 min, 37°C) or maintenance medium (negative control) (1 h, 4°C), fixed (4% PFA, 15 min, room temperature), and then incubated with anti-PEDV/PEAV N (red), and cytoskeletal changes were observed via probing actin with Alexa Fluor 488 phalloidin (green). (B) Vero cells were inoculated with PEDV/PEAV (MOI = 20) (1 h, 4°C), pretreated with PMA (positive control) (200 nM, 60 min, 37°C) or maintenance medium (negative control) (1 h, 4°C), and then incubated in maintenance medium containing 0.5 mg/mL Alexa Fluor 488-labeled dextran (15 min, 37°C), fixed (4% PFA, 15 min, room temperature), and then incubated with anti-PEDV/PEAV N (red). Dextran uptake was visualized using immunofluorescence microscopy. (C) Dextran uptake by Vero cells is represented by dextran fluorescence IDO values measured with ImageJ software. The mean ± SD values represent three individual pictures (***, P < 0.001; ns, P > 0.05).
(TIF)

S4 Fig ALIX does not interact directly with PEDV or PEAV structural proteins. (A) HEK-293T cells co-transfected with PEDV structural protein expression plasmids (HA-E, M, N, S1, and S2) and Flag-ALIX. (B) HEK-293T cells co-transfected with PEAV structural protein expression plasmids (HA-E, M, N, S1, and S2) and Flag-ALIX for 24 h. GAPDH is the loading control.
(TIF)

S5 Fig The interaction between TSG101 and PEDV/PEAV M protein. (A) Vero cells transfected with PEAV HA-M-WT, M-PVAA, or M-AVAA plasmids for 24 h; red: anti-HA, green: anti-TSG101; Scale bar = 5 μm. (B) TSG101 and PEAV M expression in HEK-293T cells co-transfected with PEAV HA-M (WT) or M (PVAA) or M (PVAA), and Flag-TSG101 for 24 h; GAPDH is the loading control. (C) HEK-293T cells transfected with structural plasmids for PEDV (HA-E, M, N, S1, and S2), and Flag-TSG101 for 24 h. The sample name is indicated above each swimlane, and the black arrow represents the migration location of positive proteins. (D) PEDV M protein truncation details. “MN” represents the N-terminus of M protein, “MC” represents the C-terminus of M protein, “NPM” represents “no proline motif”, “PM” represents “contain proline motif”, and the position of the protein amino acids is indicated in the box. (E and F) HEK-293T cells transfected with truncated PEDV or PEAV M proteins; (E) PEAV or PEDV M-N or C or (F) PEAV or PEDV M-C1 or C2, and Flag-TSG101 for 24 h. The sample name is indicated above each swimlane, and the black arrow represents the migration location of positive proteins. (G) PEAV or PEDV NPM-1 or 2 or (H) PEAV or PEDV M-PM-1 or 2, and Flag-TSG101 for 24 h. The sample name is indicated above each swimlane, and the black arrow represents the migration location of positive proteins.
(TIF)

S6 Fig Vero or jejunal epithelial cells viabilities following incubation with each drug and siRNA duplex tested in this work, as assessed using the CCK8 cell viability detection kit. All results are presented as the mean ± SD from three independent experiments (**, P<0.01).
(TIF)

S1 Table Primers used in this study. (XLSX)

S2 Table siRNA duplxes used in this study. (XLSX)

S1 Data Excel spreadsheet containing, in separate sheets, the underlying numerical data and statistical analysis graph for Figs 1A and 1B, 2C and 2D, 3B, 3C and 3F, 4C, 5B and 5C, 6D, 7C, 7D, S1D, S3C, and S6. (XLSX)
