# Supplementary figures and images for: ALIX and TSG101 are essential for cellular entry and replication of two porcine alphacoronaviruses
Source: PLoS Pathog. 2024 Mar 15;20(3):e1012103. doi: 10.1371/journal.ppat.1012103 (PMC10971774; doi:10.1371/journal.ppat.1012103)

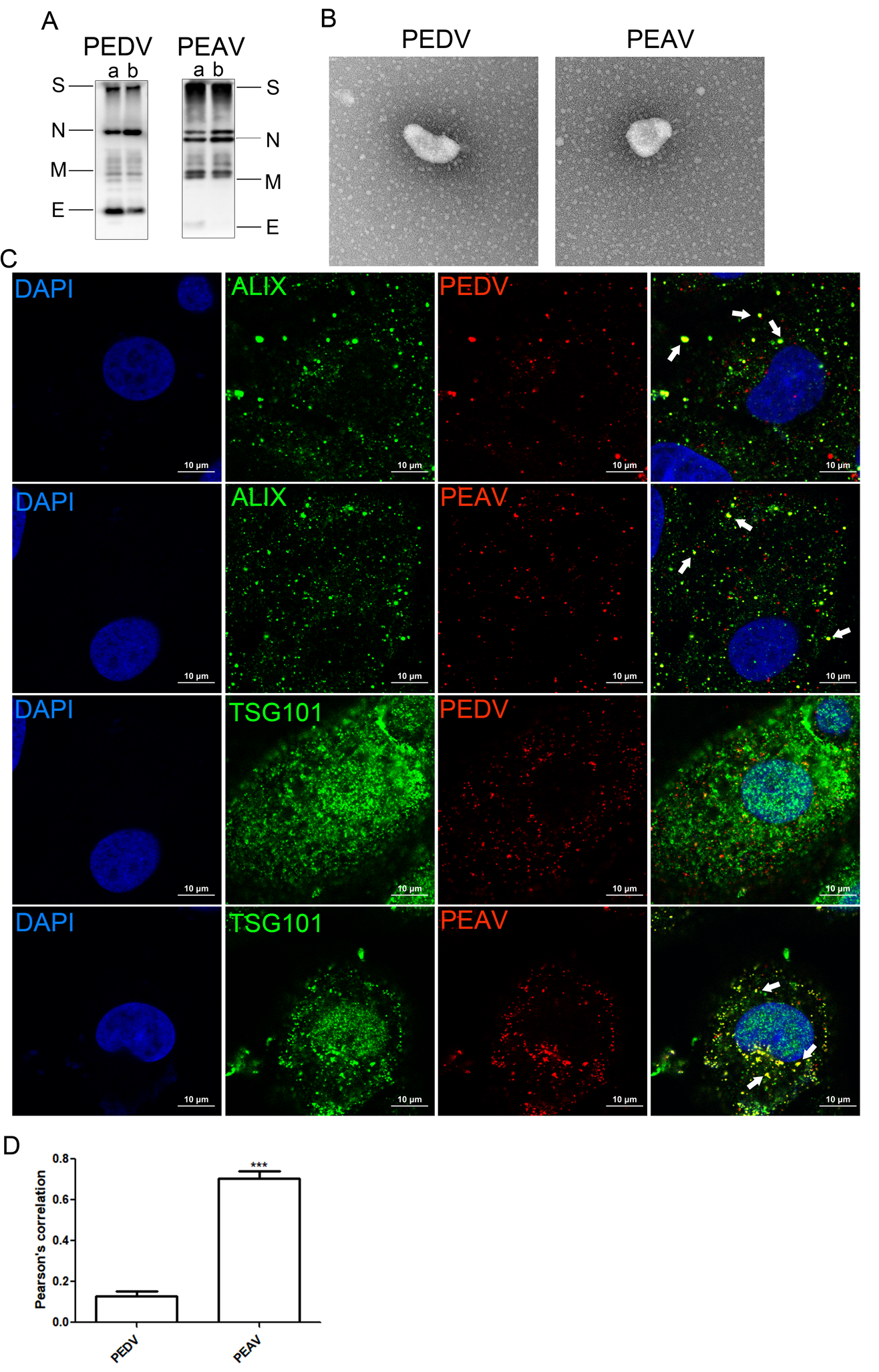

Supplement: S1 Fig — (A and B) (A) PEDV VLPs, PEAV VLPs, all of which were produced from 293T cells, were independently purified by sucrose gradient centrifugation and 5 μg of purified VLPs were applied to each lane of SDS-PAGE; a: S: N: M: E molar ratio is 1:1:1:1; b: S: N: M: E molar ratio is 1:2:1:1. (B) Negative staining of PEDV VLP, PEAV VLP. (C) Vero cells inoculated with PEDV VLP or PEAV VLP for 30 min (10 μg); red: anti-PEDV N or anti-PEAV N, green: anti-ALIX or anti-TSG101, blue: Dapi; white arrows represent co- located clusters. Scale bar = 10 μm. (D) Co-localization of TSG101 with PEDV or PEAV N expressed as Pearson’s correlation coefficient, measured for individual cells. All results are presented as the mean ± SD from three independent experiments (***, P < 0.001). (TIF) [file ppat.1012103.s001.tif]

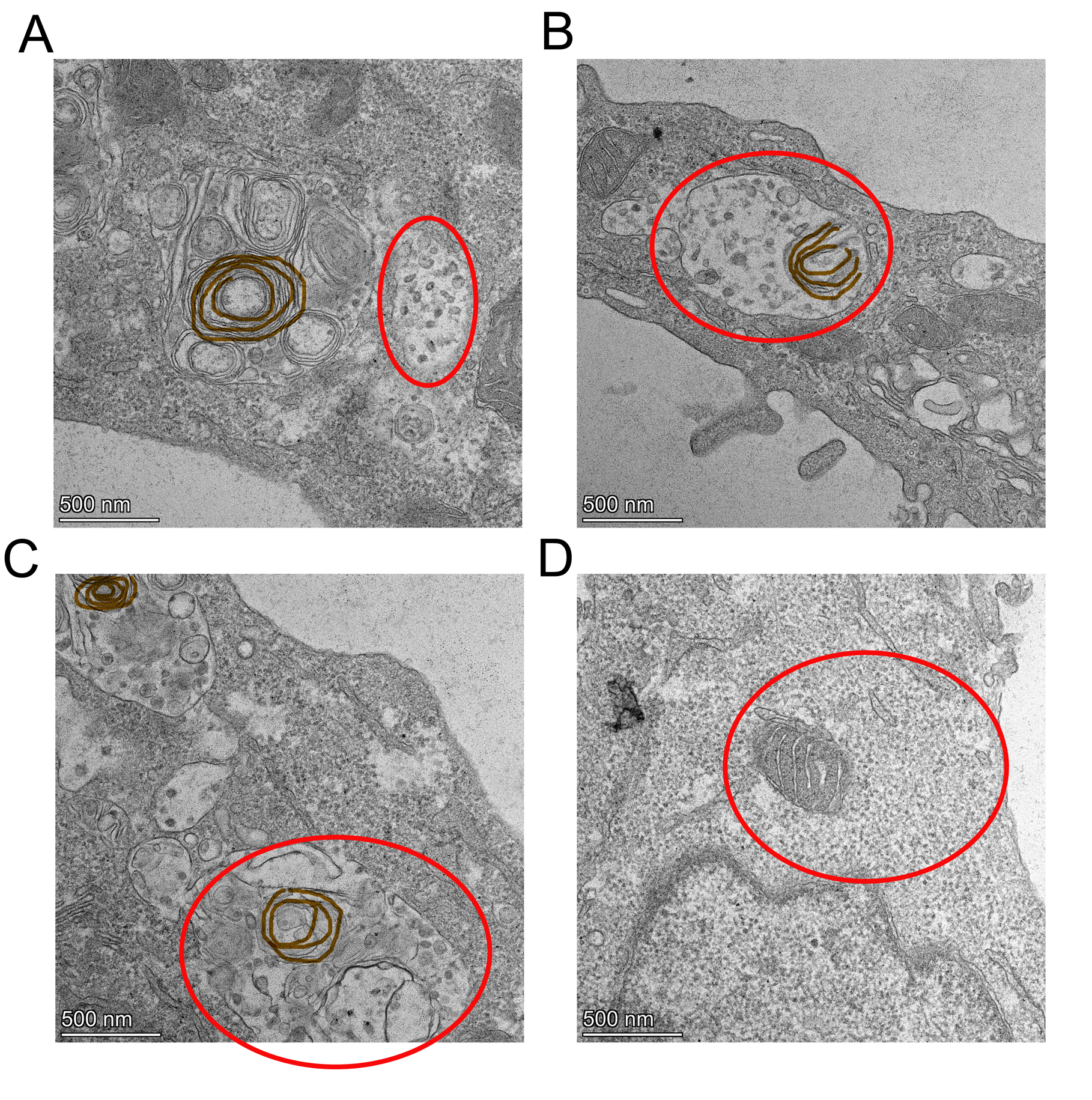

Supplement: S2 Fig — (A) The red circle represents a complete vesicles containing viruses, recorded as “+”; Scale bar = 5 μm. (B) The red circle represents a complete vesicles containing viruses and lysosomal structures, recorded as “-”; Scale bar = 5 μm. (C) Same as (B). (D) Mock-infected group. The red circle represents that there are no vesicles containing viruses or viral mixed lysosomal structures. Brown irregular lines: lysosomal structures. (TIF) [file ppat.1012103.s002.tif]

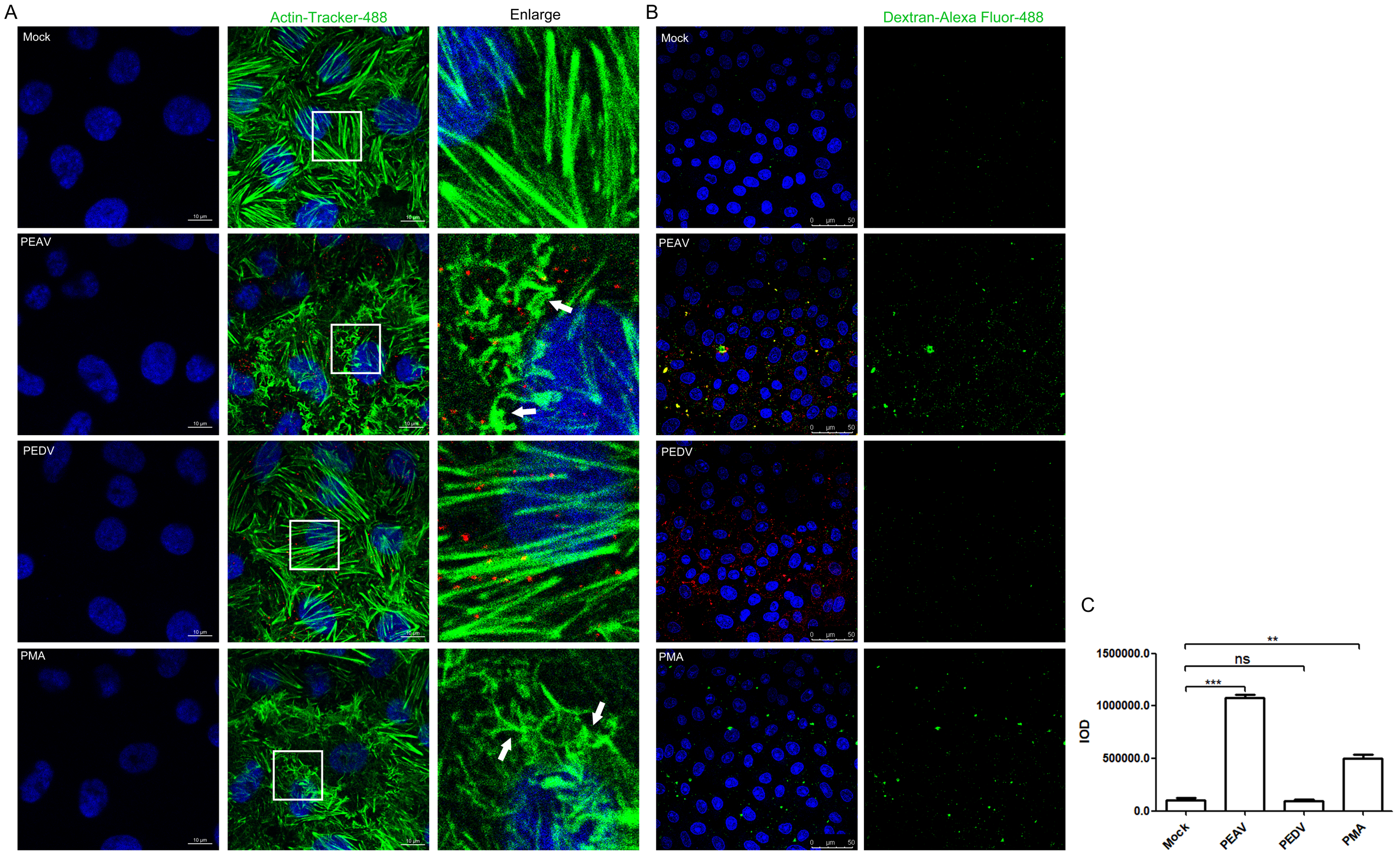

Supplement: S3 Fig — (A) Vero cells were inoculated with PEDV/PEAV (MOI = 20) (1 h, 4°C), washed, incubated (15 min, 37°C), and fixed (4% PFA, 15 min, RT). Vero cells were pretreated with PMA (positive control) (200 nM, 60 min, 37°C) or maintenance medium (negative control) (1 h, 4°C), fixed (4% PFA, 15 min, room temperature), and then incubated with anti-PEDV/PEAV N (red), and cytoskeletal changes were observed via probing actin with Alexa Fluor 488 phalloidin (green). (B) Vero cells were inoculated with PEDV/PEAV (MOI = 20) (1 h, 4°C), pretreated with PMA (positive control) (200 nM, 60 min, 37°C) or maintenance medium (negative control) (1 h, 4°C), and then incubated in maintenance medium containing 0.5 mg/mL Alexa Fluor 488-labeled dextran (15 min, 37°C), fixed (4% PFA, 15 min, room temperature), and then incubated with anti-PEDV/PEAV N (red). Dextran uptake was visualized using immunofluorescence microscopy. (C) Dextran uptake by Vero cells is represented by dextran fluorescence IDO values measured with ImageJ software. The mean ± SD values represent three individual pictures (***, P < 0.001; ns, P > 0.05). (TIF) [file ppat.1012103.s003.tif]

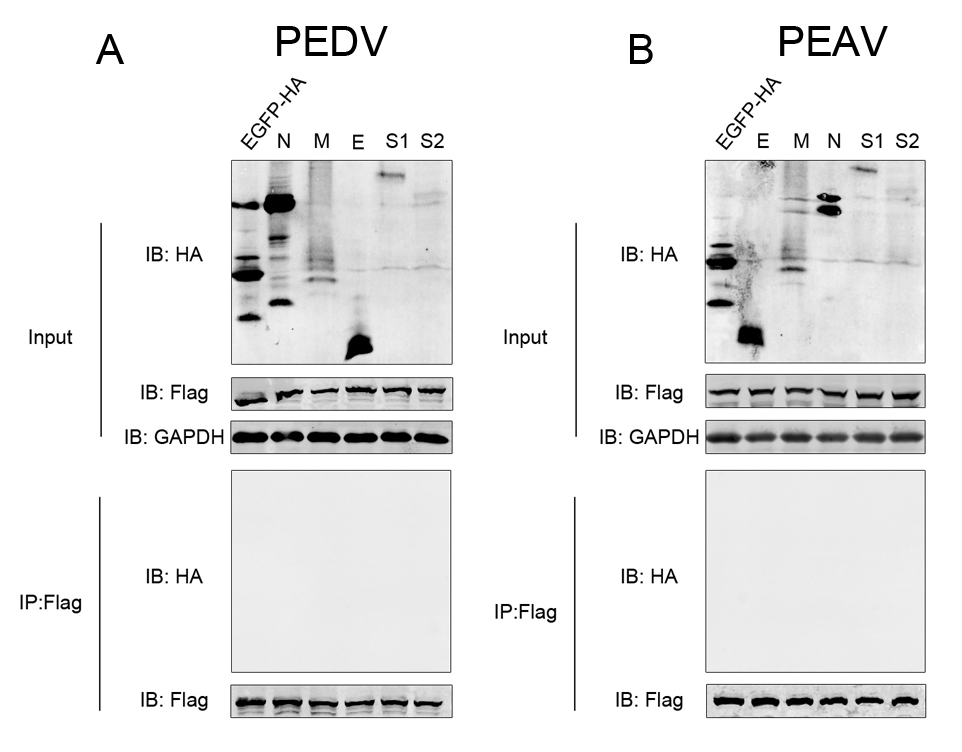

Supplement: S4 Fig — (A) HEK-293T cells co-transfected with PEDV structural protein expression plasmids (HA-E, M, N, S1, and S2) and Flag-ALIX. (B) HEK-293T cells co-transfected with PEAV structural protein expression plasmids (HA-E, M, N, S1, and S2) and Flag-ALIX for 24 h. GAPDH is the loading control. (TIF) [file ppat.1012103.s004.tif]

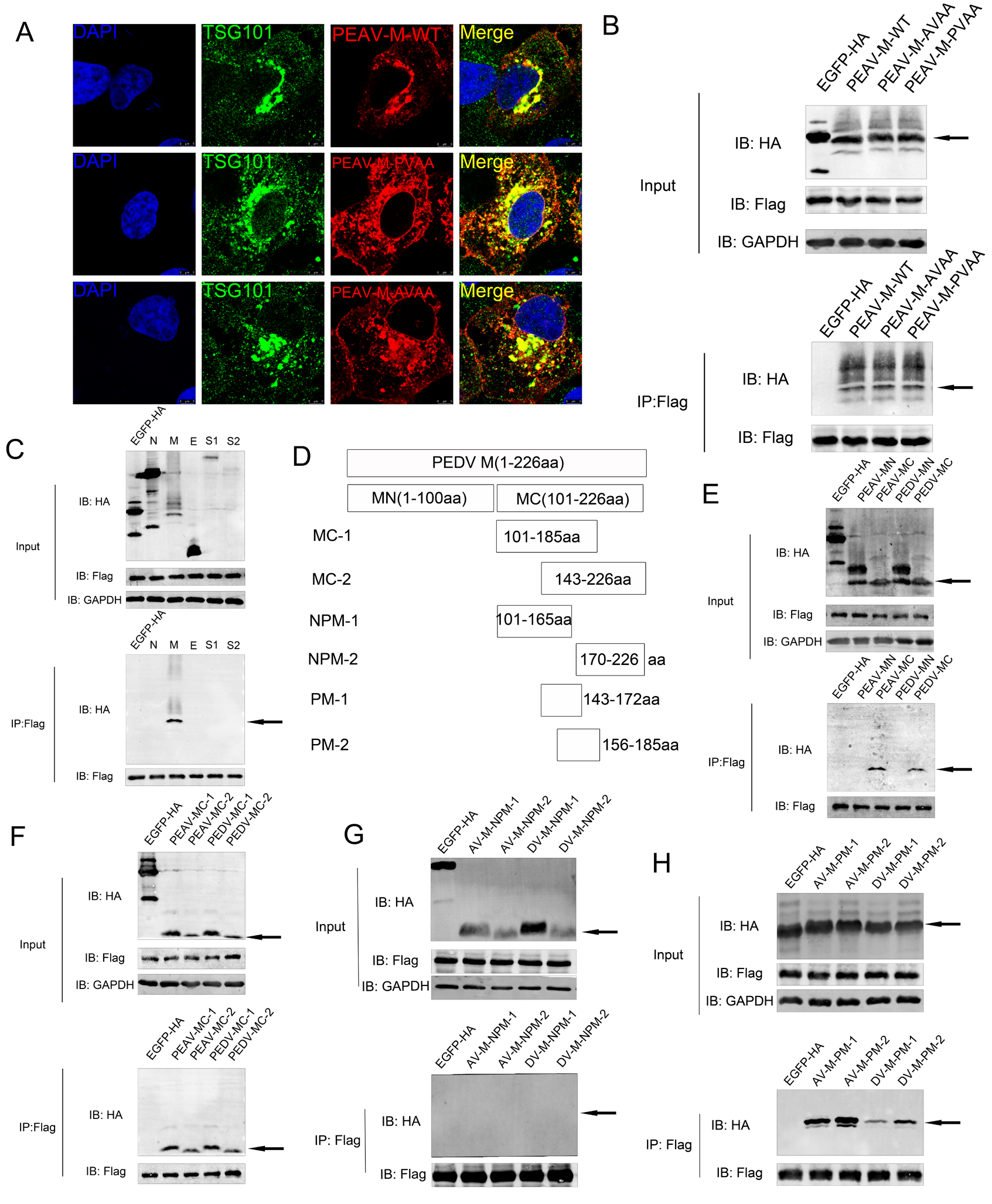

Supplement: S5 Fig — (A) Vero cells transfected with PEAV HA-M-WT, M-PVAA, or M-AVAA plasmids for 24 h; red: anti-HA, green: anti-TSG101; Scale bar = 5 μm. (B) TSG101 and PEAV M expression in HEK-293T cells co-transfected with PEAV HA-M (WT) or M (PVAA) or M (PVAA), and Flag-TSG101 for 24 h; GAPDH is the loading control. (C) HEK-293T cells transfected with structural plasmids for PEDV (HA-E, M, N, S1, and S2), and Flag-TSG101 for 24 h. The sample name is indicated above each swimlane, and the black arrow represents the migration location of positive proteins. (D) PEDV M protein truncation details. “MN” represents the N-terminus of M protein, “MC” represents the C-terminus of M protein, “NPM” represents “no proline motif”, “PM” represents “contain proline motif”, and the position of the protein amino acids is indicated in the box. (E and F) HEK-293T cells transfected with truncated PEDV or PEAV M proteins; (E) PEAV or PEDV M-N or C or (F) PEAV or PEDV M-C1 or C2, and Flag-TSG101 for 24 h. The sample name is indicated above each swimlane, and the black arrow represents the migration location of positive proteins. (G) PEAV or PEDV NPM-1 or 2 or (H) PEAV or PEDV M-PM-1 or 2, and Flag-TSG101 for 24 h. The sample name is indicated above each swimlane, and the black arrow represents the migration location of positive proteins. (TIF) [file ppat.1012103.s005.tif]

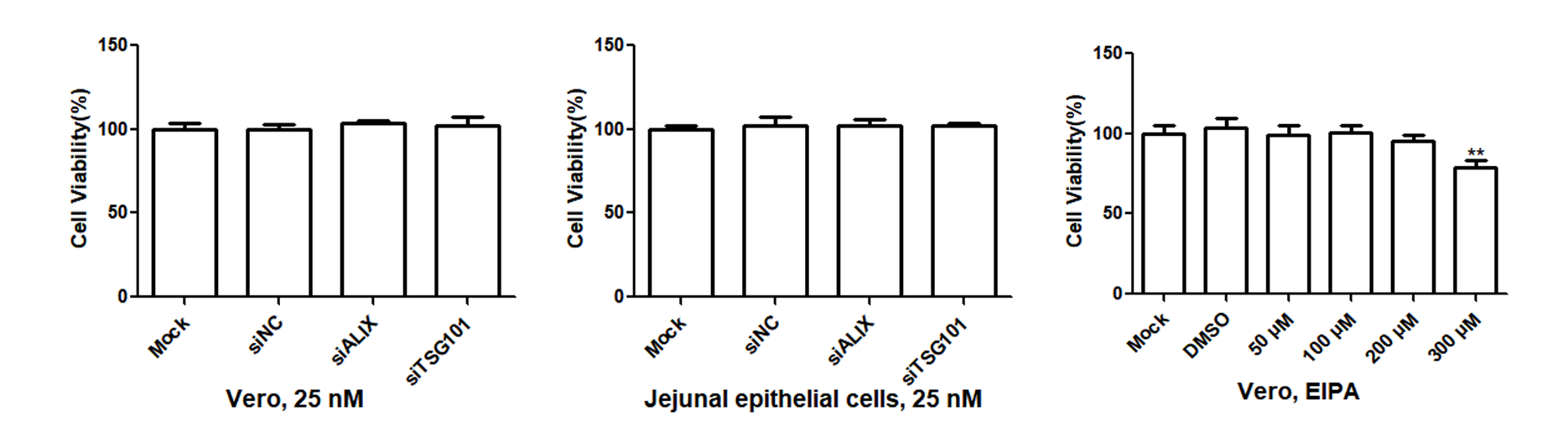

Supplement: S6 Fig — All results are presented as the mean ± SD from three independent experiments (**, P<0.01). (TIF) [file ppat.1012103.s006.tif]
